# Is Tobacco Use Associated With Risk of Recurrence and Mortality Among People With TB? A Systematic Review and Meta-Analysis

**DOI:** 10.1016/j.chest.2023.08.021

**Published:** 2023-08-29

**Authors:** Aishwarya Lakshmi Vidyasagaran, Anne Readshaw, Melanie Boeckmann, Alexander Jarde, Faraz Siddiqui, Anna-Marie Marshall, Janita Akram, Jonathan E. Golub, Kamran Siddiqi, Omara Dogar

**Affiliations:** a Department of Health Sciences, University of York, York, England; b Yorkshire and North Lincolnshire Area Team, Natural England; c Department of Global Health, Institute of Public Health and Nursing Research (IPP), University of Bremen, Bremen, Germany; d Université de Paris, Centre d’Épidémiologie Clinique, Hôpital Hôtel-Dieu, and Cochrane France, Paris, France; e Research Fellow in Public Health and lecturer in Public Health and Psychology, Helen McArdle Nursing and Care Research Institute, Faculty of Health Sciences and Wellbeing, University of Sunderland, Sunderland, England; f Hull York Medical School, University of York, York, England; g Center for Tuberculosis Research, School of Medicine, Johns Hopkins University, Baltimore, MD; h Usher Institute, The University of Edinburgh, Edinburgh, Scotland

**Keywords:** meta-analysis, risk of mortality, risk of recurrence, smoking, systematic review, tobacco, TB

## Abstract

**Background:**

Associations between tobacco use and poor TB treatment outcomes are well documented. However, for important outcomes such as TB recurrence or relapse and mortality during treatment, as well as for associations with smokeless tobacco (ST), the evidence is not summarized systematically.

**Research Question:**

Is tobacco use associated with risk of poor treatment outcomes among people with TB?

**Study Design and Methods:**

The MEDLINE, Embase, and Cumulative Index of Nursing and Allied Health Literature databases were searched on November 22, 2021. Epidemiologic studies reporting associations between tobacco use and at least one TB treatment outcome were eligible. Independent double-screening, extractions, and quality assessments were undertaken. Random effects meta-analyses were conducted for the two primary review outcomes (TB recurrence or relapse and mortality during treatment), and heterogeneity was explored using subgroups. Other outcomes were synthesized narratively.

**Results:**

Our searches identified 1,249 records, of which 28 were included in the meta-analyses. Based on 15 studies, higher risk of TB recurrence or relapse was found with ever using tobacco vs never using tobacco (risk ratio [RR], 1.78; 95% CI, 1.31-2.43; *I*^2^ = 85%), current tobacco use vs no tobacco use (RR, 1.95; 95% CI, 1.59-2.40; *I*^2^ = 72%), and former tobacco use vs never using tobacco (RR, 1.84; 95% CI, 1.21-2.80; *I*^2^ = 4%); heterogeneity arose from differences in study quality, design, and participant characteristics. Thirty-eight studies were identified for mortality, of which 13 reported mortality during treatment. Ever tobacco use (RR, 1.55; 95% CI, 1.32-1.81; *I*^2^ = 0%) and current tobacco use (RR, 1.51; 95% CI, 1.09-2.10; *I*^2^ = 87%) significantly increased the likelihood of mortality during treatment among people with TB compared with never using tobacco and not currently using tobacco, respectively; heterogeneity was explained largely by differences in study design. Almost all studies in the meta-analyses scored high or moderate on quality assessments. Narrative synthesis showed that tobacco use was a risk factor for other unfavorable TB treatment outcomes, as previously documented. Evidence on ST was limited, but identified studies suggested an increased risk for poor outcomes with its use compared with not using it.

**Interpretation:**

Tobacco use significantly increases the risk of TB recurrence or relapse and mortality during treatment among people with TB, highlighting the need to address tobacco use to improve TB outcomes.

**Trial Registry:**

PROSPERO; No.: CRD42017060821; URL: https://www.crd.york.ac.uk/prospero/

## Graphical Abstract

**Figure:**
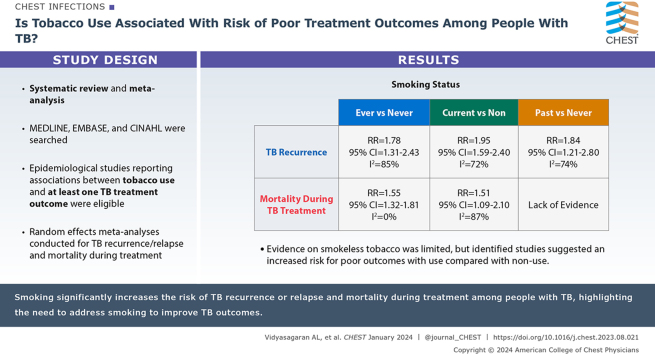

**Take-home Points**

**Study Question:** Is tobacco use associated with the risk of recurrence and mortality among people with TB?

**Results:** A higher risk of TB recurrence or relapse was found with ever using tobacco vs never using tobacco (risk ratio [RR], 1.78; 95% CI, 1.31-2.43; *I*^2^ = 85%), current tobacco use vs no tobacco use (RR, 1.95; 95% CI, 1.59-2.40; *I*^2^ = 72%), and former tobacco use vs never using tobacco (RR, 1.84; 95% CI, 1.21-2.80; *I*^2^ = 4%). Moreover, ever tobacco use (RR, 1.55; 95% CI, 1.32-1.81; *I*^2^ = 0%) and current tobacco use (RR, 1.51; 95% CI, 1.09-2.10; *I*^2^ = 87%) significantly increased the likelihood of mortality among people with TB compared with never and no tobacco use, respectively. Evidence on smokeless tobacco was limited, but some studies suggested an increased risk of poor outcomes associated with its use compared with not using it.

**Interpretation:** Tobacco use significantly increases the risk of TB recurrence or relapse and mortality during treatment among people with TB, highlighting the need to address tobacco use to improve TB outcomes.

Tobacco use and TB contribute significantly to the global burden of disease, both individually and by acting synergistically. Although global tobacco use prevalence has declined (22.7% in 2007 to 19.6% in 2019), the total number of people using tobacco remains high because of population growth.^1^ More than 80% of the 1.3 billion individuals worldwide who use tobacco live in low-income and middle-income countries (LMICs), where the TB burden also is substantial.^2^ Not only is this dual burden a grave problem in LMICs, but also tobacco use rates are estimated to be higher (approximately 8%) among people with TB than in the general population.^3^ Assuming that the relative prevalence of tobacco use and TB remain stable, it is estimated that > 40 million TB-related deaths will be attributable to tobacco use by 2050.^4^ In addition, smokeless tobacco (ST) is consumed by > 300 million people worldwide, with some studies suggesting adverse associations with TB.^5^^,^^6^ In South Asian countries, ST use tends to be even higher than tobacco use alone among people with TB.^7^

TB is one of the most common chronic infectious diseases. In 2020, approximately 1.3 million TB-related deaths occurred among people without HIV, up from 1.2 million in 2019.^8^ COVID-19 has impeded further an already fragile global response to ending TB, with the first year-on-year estimated increase since 2005 in the number of TB deaths for 2020 and 2021.^8^^,^^9^ In these challenging times, integrating policies for tobacco control within routine TB care becomes particularly critical.

Moderate to strong evidence on the association of tobacco use with TB infection (latent) and disease (active TB) exists; however, evidence on TB mortality resulting from tobacco use was inconclusive in systematic reviews last conducted in 2007.^10^, ^11^, ^12^ Although one of those reviews also reported significant association of retreatment TB with tobacco use,^12^ this finding was based on only two studies. Since then, several studies have been published on this topic. Two systematic reviews in 2020 further identified negative impacts of tobacco use on TB treatment.^13^^,^^14^ However, these reviews presented combined outcomes, one as “poor outcomes” (combining failure, loss to follow-up, and death)^13^ and the other as “unfavourable outcomes” (combining failure, transfer, loss to follow-up, and death).^14^ Both reviews did not include TB recurrence or relapse explicitly, and the latter included only current tobacco use, which limited the scope.

Given the importance of reducing TB recurrence or relapse especially in the context of drug resistance and related mortality to the End Tuberculosis Strategy,^15^ we determined their association with tobacco use. Our risk estimates offer what was missed in previous meta-analyses, including an expanded remit to include all tobacco products.

## Study Design and Methods

This review was registered with PROSPERO (Identifier: CRD42017060821) and follows the Preferred Reporting Items for Systematic Reviews and Meta-Analysis guidelines (e-Appendix 1).^16^

### Search Strategy and Selection Criteria

Three electronic databases (MEDLINE, Embase, and Cumulative Index of Nursing and Allied Health Literature) were searched from inception to November 22, 2021. Search terms for tobacco use (*smoking*, *smokeless*) were developed from previous reviews, whereas those for TB outcomes were developed from a monograph on TB and tobacco control.^17^ Searches were conducted by combining both sets of terms (e-Appendix 2); no language restrictions were applied during searching.

We included epidemiologic studies (cohort, case control, and cross-sectional) on people with TB (not restricted by age, sex, comorbidities, pulmonary or extrapulmonary presentation, or geographic region) that measured the effect of ever, current, or past tobacco use (with smoke and smokeless) on TB treatment outcomes (Table 1). Studies that included both people with drug-susceptible and drug-resistant TB (DRTB) were eligible and were analyzed as explained herein. However, studies on treatment outcomes exclusively among people with DRTB were excluded because the treatment course and its association with tobacco is likely to be different in this population. Similarly, studies on treatment outcomes exclusively among people with retreatment TB were excluded, whereas studies that included both people with new and retreatment TB were eligible. Our primary review outcomes were TB recurrence or relapse and mortality during treatment; within the outcome of mortality, we also included all-cause mortality among people with TB and TB mortality. Secondary outcomes were default, failure, unsuccessful treatment (combined mortality, default, and failure), delayed sputum conversion, treatment nonadherence, severity of disease, and drug resistance development. Studies reporting secondhand tobacco smoke exposure or unclear outcomes were excluded. Randomized controlled trials, reviews, case series, and case reports also were excluded.

**Table 1:** Definitions for TB Treatment Outcomes

Outcome	Definition
Recurrence or relapse^a^	Those previously treated for TB who were declared cured or who completed treatment at the end of the most recent course and again receive a diagnosis of an episode of TB (either a true relapse or a new episode of TB caused by reinfection, also known as recurrence).
Mortality^b^	• TB mortality: the cause of death designated as being the result of TB or dying with verified TB. Death certificate notification, medical records, or family interviews are considered acceptable sources of information. • Death during TB treatment • All-cause mortality among people with a TB diagnosis
Treatment default^a^	Those previously treated for TB who were declared lost to follow-up at the end of the most recent course of treatment
Treatment failure^a^	Those treated for TB for whom the most recent course of treatment failed
Delayed sputum conversion^a^	Delayed conversion rate of positive sputum smear results in patients with pulmonary TB at follow-up (after 2 months of therapy). Nonconversion was defined as persistent positive sputum smear results for patients with TB at the end of the 2- or 3-mo intensive phase of treatment.
Poor treatment adherence	Both compliance with the number of days anti-TB drugs were taken or the number of tablets taken of the prescribed amount is considered an acceptable measure of adherence.
Severity of disease^b^	Higher bacillary load (smear grading 3+ and higher), more cavitation (advanced radiologic lesions), hospitalized, symptoms (cough, dyspnea, upper zone involvement)
Drug-resistant TB^a^	TB that is resistant to ≥ 1 first-line antituberculosis drugs

a World Health Organization Global Tuberculosis Report, 2013.^18^

b TB/tobacco monograph.^17^

We screened the references of included articles and relevant systematic reviews to identify additional studies. All identified reports underwent deduplication and independent double screening by two of the authors (A. R. and M. B.) based on title and abstract. Full-text review of potentially relevant articles also was assessed independently by two reviewers (F. S. and M. B.), whereas a third reviewer (A. L. V. or O. D.) was consulted when consensus could not be reached. During screening, we considered only studies written in English because of constrained resources for translation.

### Data Extraction and Synthesis

Groups of two reviewers (A. R. and A.-M. M. or A. J. and A. L. V.) independently extracted data from included studies using a piloted data extraction form specifically designed for this review. The main sections included: study design and characteristics; sample size and participant demographics; and exposure and outcome details, including type of tobacco (with smoke or smokeless), type of exposure (ever, current, or past), frequency of outcome among exposed and unexposed participants, and the measures of effect reported. The extraction forms were compared, and disagreements were resolved in the first instance by discussion or with a third reviewer (O. D.) if consensus could not be reached.

Risk of bias was evaluated using the Quality Assessment Tool for Quantitative Studies,^19^ and each study was rated as strong, moderate, or weak in the following categories: study design, analysis, withdrawals and dropouts, data collection, selection bias, and confounders. Based on these, an overall rating was provided. Subsequently, we considered the influence of studies with weak methodologic quality on summary effect sizes.

Meta-analysis was carried out using RevMan version 5.4 software (Cochrane Collaboration).^20^ We classified the studies according to tobacco type (with smoke or smokeless) and exposure type (ever vs never use or current use vs current nonuse or past use vs never use) for each treatment outcome and performed meta-analysis for groups that included two or more studies. We limited the meta-analysis to our two primary outcomes (TB recurrence or relapse and mortality during treatment) and narratively synthesized the additional outcomes because they largely were covered in two reviews published in 2020.^13^^,^^14^

For the meta-analyses, we used the number of individuals exposed, number of individuals unexposed, and events observed in both those groups as reported in the individual studies to calculate risk ratios (RRs) and 95% CIs. These estimates were pooled using random effects models and are presented as forest plots. Heterogeneity of included studies was assessed using the *I*^2^ statistic, and the reasons for heterogeneity were explored through subgroup analyses according to study design, quality, and presence of comorbidities among participants. In addition, sensitivity analyses were performed by removing (1) studies that included people with DRTB and (2) studies that included people with retreatment TB. Finally, the presence of publication bias was assessed based on funnel plots, and Grading of Recommendations, Assessment, Development, and Evaluations (GRADE) assessments were used to rate the certainty of evidence for the primary outcomes.^21^ We did not explore the dose-response effect of duration and amount of tobacco use on TB outcomes.

## Results

**Figure 1:**
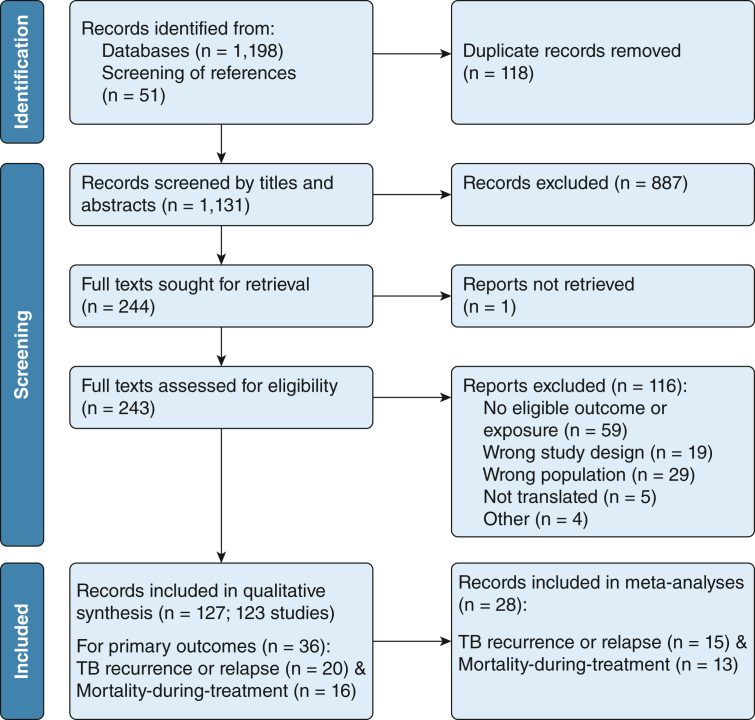
Preferred Reporting Items for Systematic Reviews and Meta-Analyses flow chart showing study selection.

Our searches retrieved 1,249 records. After removing duplications, 1,131 records were screened on titles and abstracts, and 887 records were excluded. Of the remaining 244 records, we retrieved and screened 243 full texts and excluded an additional 116 records (Fig 1, e-Appendix 3).

In total, 127 records were included (Table 2).^22^, ^23^, ^24^, ^25^, ^26^, ^27^, ^28^, ^29^, ^30^, ^31^, ^32^, ^33^, ^34^, ^35^, ^36^, ^37^, ^38^, ^39^, ^40^, ^41^, ^42^, ^43^, ^44^, ^45^, ^46^, ^47^, ^48^, ^49^, ^50^, ^51^, ^52^, ^53^, ^54^, ^55^, ^56^, ^57^, ^58^, ^59^, ^60^, ^61^, ^62^, ^63^, ^64^, ^65^, ^66^, ^67^, ^68^, ^69^, ^70^, ^71^, ^72^, ^73^, ^74^, ^75^, ^76^, ^77^, ^78^, ^79^, ^80^, ^81^, ^82^, ^83^, ^84^, ^85^, ^86^, ^87^, ^88^, ^89^, ^90^, ^91^, ^92^, ^93^, ^94^, ^95^, ^96^, ^97^, ^98^, ^99^, ^100^, ^101^, ^102^, ^103^, ^104^, ^105^, ^106^, ^107^, ^108^, ^109^, ^110^, ^111^, ^112^, ^113^, ^114^, ^115^, ^116^, ^117^, ^118^, ^119^, ^120^, ^121^, ^122^, ^123^, ^124^, ^125^, ^126^, ^127^, ^128^, ^129^, ^130^, ^131^, ^132^, ^133^, ^134^, ^135^, ^136^, ^137^, ^138^, ^139^, ^140^, ^141^, ^142^, ^143^, ^144^, ^145^, ^146^, ^147^, ^148^ They comprised 123 unique studies; four studies were reported in two publications each.^24^^,^^25^^,^^62^^,^^63^^,^^82^^,^^83^^,^^123^^,^^124^ For the two primary outcomes, we included 36 studies (20 on recurrence or relapse^36^^,^^39^^,^^43^^,^^47^^,^^51^^,^^64^^,^^67^^,^^71^^,^^84^^,^^88^^,^^94^^,^^96^^,^^97^^,^^100^^,^^109^^,^^112^^,^^118^^,^^123^^,^^127^^,^^147^ and 16 on mortality during treatment.^32^^,^^54^^,^^55^^,^^57^^,^^66^^,^^70^^,^^74^^,^^90^^,^^95^^,^^99^^,^^103^^,^^111^^,^^120^^,^^125^^,^^126^^,^^135^ However, data from eight studies could not be pooled (five on recurrence or relapse^39^^,^^47^^,^^67^^,^^123^^,^^127^ and three on mortality^55^^,^^66^^,^^90^) because the numbers required for computing RRs were not reported. Therefore, 15 studies were included in the TB recurrence or relapse meta-analyses and 13 studies were included in the mortality during treatment meta-analyses (e-Table 1).

**Table 2:** Summary of Included Studies

Reference	Study Year	Country, Region	Study Design	Sample Size	Age; Male Sex (%); Habits, Comorbidities	PTB/EPTB; DRTB; Retreatment	Exposure(s)	Outcome(s)	Study Quality
Liu et al^22^ (1998)	1989—1991	China, WPR	Retrospective cohort	1 million	35+; NR; NR	PTB; yes; NR	Current tobacco use	TB mortality	Strong
Lam et al^23^ (2001)	1997—1999	Hong Kong, WPR	Case-control	40,561	35+; 47.4%; NR	PTB; NR; NR	Past tobacco use	TB mortality	Moderate
Leung et al^24^ (2002) Leung et al^25^ (2003)	1996	Hong Kong, WPR	Retrospective cohort	893	16+; > 68.0%; Chronic disease	PTB and EPTB; yes (4.9%); yes	Ever tobacco use	Unsuccessful, delayed conversion	Moderate
Santha et al^26^ (2002)	1999—2000	India, SEAR	Retrospective cohort	676	14-87; 75.0%; Alcohol use	PTB and EPTB; yes; yes	Current tobacco use	Failure, default	Moderate
Gajalakshmi et al^27^ (2003)	1995—2000	India, SEAR	Case-control	35,000	25-69; 100%; NR	PTB; yes; yes	Ever tobacco use (cigarette, bidi)	TB mortality	Strong
Salami and Oluboyo^28^ (2003)	1991—1999	Nigeria, AFR	Retrospective cohort	1,741	15+; 45.6%; alcohol use, HIV, chronic disease	PTB; NR; no	Current tobacco use	Default	Moderate
Chang et al^29^ (2004)	1999—2001	Hong Kong, WPR	Case-control (nested)	408	15+; 86.3%; alcohol and drug use, HIV, hepatitis	PTB; yes; yes	Ever, current, past tobacco use	Default	Strong
Sitas et al^30^ (2004)	1994—1998	South Africa, AFR	Case-control	5,340	25+; NR; NR	NR	Ever tobacco use	TB mortality	Strong
Abal et al^31^ (2005)	1998—2000	Kuwait, EMR	Prospective cohort	339	Adults; 78.8%; alcohol and drug use, DM	PTB; no; NR	Current tobacco use	Delayed conversion	Moderate
Altet-Gomez et al^32^ (2005)	1996—2002	Spain, EUR	Retrospective cohort	13,038	14+; 67.6%; alcohol and drug use, HIV	PTB and EPTB; NR; NR	Current tobacco use	On-treatment mortality, severity	Strong
Balbay et al^33^ (2005)	1998—2003	Turkey, EUR	Retrospective cohort	154	16-82; 65.6%; alcohol use	PTB and EPTB; no; yes	Current tobacco use	Nonadherence	Strong
Chandrasekaran et al^34^ (2005)	1999—2002	India, SEAR	Prospective cohort	1,406	All ages; 69.5%; alcohol use	PTB; NR; no	Current tobacco use	Default	Moderate
Gupta et al^35^ (2005)	1992—1999	India, SEAR	Prospective cohort	99,570	35+; NR; NR	PTB; NR; NR	Ever tobacco use (cigarette, bidi) and smokeless	TB mortality	Strong
Thomas et al^36^ (2005)	2000—2001	India, SEAR	Prospective cohort	534	All ages; 71.2%; alcohol use	PTB; yes (5.6%); no	Current tobacco use	Recurrence or relapse	Strong
Kolappan et al^37^ (2006)	2000—2003	India, SEAR	Retrospective cohort	1,800	15+; 100%; alcohol use	PTB and EPTB; yes; yes	Current tobacco use (cigarette, bidi)	All-cause mortality	Moderate
Babb et al^38^ (2007)	2003—2004	South Africa, AFR	Retrospective cohort	220	18-65; NR; excluded people with HIV	PTB; no; no	Current tobacco use	Delayed conversion	Strong
Cacho et al^39^ (2007)	1992—2004	Spain, EUR	Retrospective cohort	645	Mean 38.6 ± 9.5; 75.0%; alcohol and drug use, HIV	PTB and EPTB; NR; NR	Current tobacco use	Recurrence/relapse	Strong
Guler et al^40^ (2007)	2000—2005	Turkey, EUR	Retrospective cohort	306	17-85; 63.1%; DM, excluded people with HIV	PTB; yes; no	Current tobacco use	Delayed conversion	Weak
Jakubowiak et al^41^ (2007)	2003	Russia, EUR	Case-control	1,805	16+; 73.0%; alcohol and drug use	PTB; no; no	Current tobacco use	Default	Moderate
Wang et al^42^ (2007)	2002—2003	Taiwan, WPR	Retrospective cohort	523	All ages; NR; alcohol use, chronic disease	PTB and EPTB; yes; no	Ever tobacco use	All-cause mortality, failure, default	Strong
d’Arc Lyra Batista et al^43^ (2008)	2001—2006	Brazil, AMR	Prospective cohort	711	13+; 64.5%; alcohol use, HIV	PTB and EPTB; NR; no	Current tobacco use	Recurrence or relapse	Strong
Jha et al^44^ (2008)	2001—2003	India, SEAR	Case-control	152,058	30-69; NR; NR	PTB and EPTB; NR; NR	Current tobacco use (cigarette, bidi) and smokeless	TB mortality	Strong
Pinidiyapathirage et al^45^ (2008)	2001—2002	Sri Lanka, SEAR	Prospective cohort	892	All ages; 74.5%; alcohol and drug use	PTB and EPTB; NR; yes	Current tobacco use	Default	Moderate
Vasantha et al^46^ (2008)	1999—2004	India, SEAR	Retrospective cohort	3,818	All ages; 73.0%; alcohol use	NR; yes; yes	Current tobacco use	On-treatment mortality	Moderate
Jee et al^47^ (2009)	1992—2006	South Korea, WPR	Prospective cohort	1,294,504	30-95; NR; alcohol use	PTB; NR; yes	Current, past tobacco use	Recurrence or relapse, TB mortality	Strong
Jiang et al^48^ (2009)	1989—1991	China, WPR	Case-control	64,899	40+; NR; NR	PTB; NR; NR	Ever tobacco use (cigarette, noncigarette)	TB mortality	Strong
Kherad et al^49^ (2009)	1999—2002	Switzerland, EUR	Retrospective cohort	252	15-92; 47.0%; alcohol and drug use, HIV	PTB and EPTB; yes; yes	Current tobacco use	Unsuccessful	NA
Kittikraisak et al^50^ (2009)	2005—2006	China, WPR	Prospective cohort	554	18+; 69.0%; alcohol and drug use, 100% HIV	PTB and EPTB; yes; NR	Current tobacco use	Default	Strong
Millet et al^51^ (2009)	1995—2005	Spain, EUR	Retrospective cohort	681	Median, 36; 67.7%; alcohol and drug use, HIV	PTB and EPTB; yes; no	Current tobacco use	Recurrence or relapse	Moderate
Metanat et al^52^ (2010)	2005—2006	Iran, EMR	Case-control	200	18+; 59.5%; comorbidities excluded	PTB; no; no	Current, past tobacco use	Delayed conversion	Weak
Siddiqui et al^53^ (2010)	2007—2008	Ireland, EUR	Retrospective cohort	53	Adults; NR; comorbidities excluded	PTB; no; NR	Ever tobacco use	Delayed conversion	Moderate
Silva et al^54^ (2010)	2005—2007	Brazil, AMR	Retrospective cohort	311	All ages; NR; HIV	PTB and EPTB; NR; no	Current tobacco use	On-treatment mortality	Strong
Tabarsi et al^55^ (2010)	2003—2009	Iran, EMR	Retrospective cohort	1,897	Mean, 50.2 ± 21.1; 51.3%; HIV	PTB; yes; yes	Current tobacco use	On-treatment mortality	NA
Vijay et al^56^ (2010)	2004—2005	India, SEAR	Case-control (nested)	1,374	15+; NR; alcohol use	PTB; NR; no	Current tobacco use	Default	Strong
Dujaili et al^57^ (2011)	2006—2008	Malaysia, WPR	Retrospective cohort	524	15+; 70.4%; alcohol and drug use, chronic disease	NR; NR; no	Ever tobacco use	On-treatment mortality, failure, default	Strong
Garcia-Garcia et al^58^ (2011)	2006—2007	Spain, EUR	Prospective cohort	1,490	18+; 61.7%; alcohol and drug use, HIV	PTB and EPTB; yes; yes	Ever tobacco use	All-cause mortality, default	NA
Maruza et al^59^ (2011)	2007—2009	Brazil, AMR	Prospective cohort	273	18-67; 69.7%; alcohol and drug use, 100% HIV	PTB and EPTB; NR; NR	Current, past tobacco use	Default	Strong
Nik Mahdi et al^60^ (2011)	2006—2007	Malaysia, WPR	Retrospective cohort	472	Mean 45.0 ± 17.9; 66.9%; HIV, DM	PTB; NR; Yes	Current tobacco use	Unsuccessful	Moderate
Solliman et al^61^ (2011)	2008—2009	Libya, EMR	Retrospective cohort	327	NR	PTB; NR; NR	Current tobacco use	Unsuccessful	NA
Tachfouti et al^62^ (2011) Tachfouti et al^63^ (2013)	2004—2009	Morocco, EMR	Prospective cohort	1,039	18-79; 95.7%; alcohol use	PTB and EPTB; NR; no	Current tobacco use	Failure, default	Strong
Anaam et al^64^ (2012)	2007—2008	Yemen, EMR	Case-control (nested)	220	15+; 58.0%; Khat use, DM	PTB; NR; no	Current tobacco use	Recurrence or relapse	Moderate
Chiang et al^65^ (2012)	2001—2003	Taiwan, WPR	Retrospective cohort	302	Adults; 68.9%; chronic disease, not HIV	PTB and EPTB; no; no	Ever tobacco use	Unsuccessful	Strong
Feng et al^66^ (2012)	2007—2009	Taiwan, WPR	Prospective cohort	1,059	Mean, 64.7 ± 19.2; 77.3%; HIV, chronic disease	PTB and EPTB; yes; no	Current tobacco use	On-treatment mortality, delayed conversion	Moderate
Lisha et al^67^ (2012)	2008—2010	India, SEAR	Retrospective cohort	224	15-80; 81.0%; alcohol and drug use, DM	PTB; yes; no	Current tobacco use	Recurrence, all-cause mortality, failure, default	NA
Tabarsi et al^68^ (2012)	2004—2007	Iran, EMR	Retrospective cohort	111	22-70; 96.3%; alcohol and drug use, 100% HIV	PTB and EPTB; NR; yes	Current tobacco use	Unsuccessful, all-cause mortality	Moderate
Visser et al^69^ (2012)	2005—2008	South Africa, AFR	Prospective cohort	113	22-43; 69.9%; alcohol use, HIV	PTB; yes; no	Ever tobacco use	Delayed conversion	Strong
Alavi-Naini et al^70^ (2013)	2002—2011	Iran, EMR	Retrospective cohort	715	15+; 52.4%; alcohol and drug use, HIV, chronic disease	PTB; NR; yes	Current tobacco use	On-treatment mortality	Strong
Bonacci et al^71^ (2013)	1995—2010	Mexico, AMR	Prospective cohort	1,062	15+; 59.0%; alcohol and drug use, DM	PTB; yes; yes	Current tobacco use	Unsuccessful, recurrence or relapse	Strong
Maciel et al^72^ (2013)	2002—2006	Brazil, AMR	Case-control (nested)	293	18-60; 66.0%; alcohol use	PTB; no; no	Current, past tobacco use	Delayed conversion	Strong
Mnisi et al^73^ (2013)	2007—2010	South Africa, AFR	Retrospective cohort	202	21-72; 98.0%; HIV	PTB and EPTB; yes; yes	Current tobacco use	Unsuccessful	Moderate
Reddy et al^74^ (2013)	2009	India, SEAR	Prospective cohort	413	15+; 81.3%; alcohol use, HIV, DM	PTB; NR; no	Current tobacco use	On-treatment mortality, failure, default	Moderate
Reed et al^75^ (2013)	NR	South Korea, WPR	Prospective cohort	657	20+; 84.0%; alcohol use, DM	PTB; yes; yes	Current tobacco use	TB mortality	Strong
Slama et al^76^ (2013)	2009—2010	Morocco, EMR	Case-control	320	15+; 80.6%; alcohol use	PTB and EPTB; NR; yes	Current tobacco use	Default	Strong
Ahmad and Velhal^77^ (2014)	2006—2007	India, SEAR	Prospective cohort	281	All ages; 74.5%; NR	PTB; NR; no	Current tobacco use and smokeless	Nonadherence	Moderate
Alo et al^78^ (2014)	2010—2012	Fiji, WPR	Retrospective cohort	395	All ages; 57.2%; DM, hypertension	PTB and EPTB; NR; yes	Current tobacco use	Unsuccessful	Moderate
Cherkaoui et al^79^ (2014)	2010—2011	Morocco, EMR	Case-control	277	Adults; 66.0%; alcohol and drug use, HIV, DM	PTB and EPTB; yes; yes	Current tobacco use	Default	Strong
Choi et al^80^ (2014)	2005—2012	South Korea, WPR	Prospective cohort	663	20+; 84.9%; alcohol and drug use, DM	PTB; yes; yes	Current tobacco use	Unsuccessful, default	Moderate
de Boer et al^81^ (2014)	2007—2009	Brazil, AMR	Prospective cohort	89	NR; 85.4%; alcohol and drug use, HIV, DM	PTB; no; NR	Current, past tobacco use	Delayed conversion	Strong
Ibrahim et al^82^ (2014) Ibrahim et al^83^ (2015)	2011—2012	Nigeria, AFR	Cross-sectional	378	15+; 60.6%; Alcohol use, HIV	PTB; NR; yes	Current tobacco use	Nonadherence, failure, default	Moderate
Louwagie and Ayo-Yusuf^84^ (2014)	2011—2013	South Africa, AFR	Cross-sectional	1,926	18+; 52.3%; alcohol and drug use, HIV	NR	Current tobacco use	Recurrence or relapse	Moderate
Lucenko et al^85^ (2014)	2006—2010	Latvia, EUR	Retrospective cohort	2,476	15+; 69.0%; alcohol and drug use, HIV	PTB and EPTB; no; no	Current tobacco use	Unsuccessful	Moderate
Pefura-Yone et al^86^ (2014)	2009—2012	Cameroon, AFR	Prospective cohort	953	15+; NR; alcohol and drug use, HIV, DM	PTB; yes; NR	Current tobacco use	Delayed conversion	Strong
Przybylski et al^87^ (2014)	2001—2010	Poland, EUR	Retrospective cohort	2,025	16-98; 67.0%; alcohol and drug use, HIV	PTB and EPTB; NR; no	Current tobacco use	Unsuccessful, adverse reaction to TB drugs	Moderate
Yen et al^88^ (2014)	2005—2011	Taiwan, WPR	Retrospective cohort	5,567	18+; 62.9%; alcohol use, HIV, cancer	PTB and EPTB; NR; NR	Current tobacco use	Recurrence or relapse	Strong
Chuang et al^89^ (2015)	2010—2012	Taiwan, WPR	Case-control	359	16+; > 66.0%; Alcohol use	PTB; NR; NR	Current, past tobacco use	Delayed conversion	Strong
Driessche et al^90^ (2015)	NR	DRC, AFR	Prospective cohort	533	Median, 38; 39.1%; alcohol and drug use, 100% HIV	PTB and EPTB; NR; NR	Ever tobacco use	On-treatment mortality, default, unsuccessful	Strong
Gegia et al^91^ (2015)	2011—2013	Georgia, EUR	Prospective cohort	524	18+; 87.2%; alcohol and drug use, HIV	PTB; yes; NR	Current, past tobacco use and smokeless	Unsuccessful	Strong
Kanda et al^92^ (2015)	2000—2002	Japan, WPR	Retrospective cohort	86	20-80; 69.8%; alcohol use, DM, excluded people with HIV	PTB; no; no	Ever tobacco use	Delayed conversion	Strong
Khan et al^93^ (2015)	2009—2010	Pakistan, EMR	Retrospective cohort	472	15+; 50.4%; HIV, DM, hepatitis	PTB and EPTB; NR; NR	Current tobacco use	Failure	Moderate
Leung et al^94^ (2015)	2001—2012	Hong Kong, WPR	Prospective cohort	16,345	All ages; NR; alcohol and drug use, HIV, DM	PTB and EPTB; yes (3.1%); yes	Current, past tobacco use	Unsuccessful, all-cause mortality, default, delayed conversion, recurrence	Strong
Liew et al^95^ (2015)	2012—2013	Malaysia, WPR	Retrospective cohort	21,582	All ages; 65.1%; HIV, DM	PTB and EPTB; yes (0.3%); yes	Current tobacco use	Unsuccessful, all-cause mortality	Strong
Mahishale et al^96^ (2015)	2012—2013	India, SEAR	Prospective cohort	2,350	15+; 74.8%; comorbidities excluded	PTB; NR; no	Current, past tobacco use (bidi, cigarette)	Recurrence or relapse	Strong
Moosazadeh et al^97^ (2015)	2002—2013	Iran, EMR	Retrospective cohort	1,271	15+; 56.2%; DM	PTB; NR; NR	Current tobacco use	Recurrence or relapse	Strong
Roy et al^98^ (2015)	2009—2011	India, SEAR	Case-control	158	Median 40; 63.3%; alcohol use	PTB; NR; no	Current tobacco use	Default	Strong
Yamana et al^99^ (2015)	2010—2013	Japan, WPR	Retrospective cohort	877	All ages; 64.5%; chronic disease	PTB; yes; no	Current tobacco use	On-treatment mortality	Strong
Ahmad et al^100^ (2016)	2015—2016	Pakistan, EMR	Case-control	332	> 10 y; 100%; comorbidities excluded	PTB; yes; no	Ever tobacco use (any form)	Recurrence or relapse	Strong
Ajili et al^101^ (2016)	NR	Tunisia, EMR	Retrospective cohort	355	All ages; NR; alcohol and drug use, chronic disease	PTB; NR; NR	Current tobacco use	Delayed conversion	NA
Rathee et al^102^ (2016)	2010—2011	India, SEAR	Prospective cohort	101	18-65; 65.3%; NR	PTB; no; NR	Current, past tobacco use (cigarette, bidi)	Default	Moderate
Rodrigo et al^103^ (2016)	2006—2013	Spain, EUR	Prospective cohort	5,182	18+; 62.0%; alcohol and drug use, HIV	PTB and EPTB; yes (6.9%); NR	Current tobacco use	On-treatment mortality	Moderate
Veerakumar et al^104^ (2016)	2013—2014	India, SEAR	Cross-sectional	235	15+; 79.6%; alcohol use	PTB; NR; yes	Current tobacco use and smokeless	Unsuccessful	Strong
Yen et al^105^ (2016)	2011—2012	Taiwan, WPR	Retrospective cohort	1,608	18+; 67.5%; alcohol use, HIV, chronic disease	PTB and EPTB; NR; yes	Current, past tobacco use	All-cause mortality	Strong
Altet et al^106^ (2017)	2013—2014	Spain, EUR	Prospective cohort	525	Mean, 34.0 ± 13.2; 62.1%; alcohol and drug use, HIV	PTB; yes; NR	Current tobacco use	Delayed conversion	Strong
Balian et al^107^ (2017)	2014—2016	Armenia, EUR	Retrospective cohort	992	Mean, 42.0 ± 17.5; 74.8%; alcohol use, HIV	PTB and EPTB; no; no	Current tobacco use	Unsuccessful	Strong
Jaber et al^108^ (2017)	2014—2015	Yemen, EUR	Prospective cohort	273	15+; 54.9%; Khat use, chronic disease	PTB; no; no	Current tobacco use	Unsuccessful, prolonged treatment duration	Strong
Kalema et al^109^ (2017)	2008—2013	Uganda, AFR	Retrospective cohort	234	18+; 58.6%; HIV	PTB; yes (3.0%); no	Ever tobacco use	Recurrence or relapse	Weak
Musteikiene et al^110^ (2017)	2015—2016	Lithuania, EUR	Prospective cohort	52	Adults; 76.9%; alcohol use, comorbidities excluded	PTB; no; no	Current tobacco use	Delayed conversion	Strong
Nagu et al^111^ (2017)	2014—2015	Tanzania, AFR	Prospective cohort	253	18+; 66.4%; alcohol and drug use, HIV, DM	PTB and EPTB; no; NR	Ever tobacco use	On-treatment mortality	Strong
Shamaei et al^112^ (2017)	2009—2012	Iran, EMR	Case-control	447	14+; > 51.0%; alcohol and drug use, HIV, chronic disease	PTB and EPTB; yes; yes	Current tobacco use	Recurrence or relapse	Moderate
Tola et al^113^ (2017)	2014	Ethiopia, AFR	Cross-sectional	698	18-90; 57.4%; alcohol and drug use, HIV	PTB and EPTB; yes (9.6%); yes	Current tobacco use	Nonadherence	Weak
Cailleaux-Cezar et al^114^ (2018)	2004—2012	Brazil, AMR	Retrospective cohort	174	Adults; 66.0%; alcohol use, DM, cancer, chronic disease	PTB; no; no	Current tobacco use	Unsuccessful, delayed conversion	Strong
Dizaji et al^115^ (2018)	2005—2015	Iran, EMR	Retrospective cohort	2,299	Adults; 50.0%; alcohol and drug use, HIV, chronic disease	PTB and EPTB; NR; no	Current tobacco use	TB mortality	Moderate
Madeira et al^116^ (2018)	2014	Brazil, AMR	Case-control	478	18+; 59.2%; alcohol and drug use, HIV, DM	PTB; no; yes	Ever tobacco use	Nonadherence	Strong
Mukhtar and Butt^117^ (2018)	2013—2014	Pakistan, EMR	Prospective cohort	614	15+; 51.0%; alcohol and drug use, DM	PTB; no; no	Current tobacco use	Unsuccessful	Strong
Rosser et al^118^ (2018)	1994—2014	UK, EUR	Case-control (nested)	246	Adults; 51.2%; alcohol use, chronic disease	PTB and EPTB; yes; no	Current tobacco use	Recurrence or relapse	Strong
Aguilar et al^119^ (2019)	2007—2015	Brazil, AMR	Case-control	284	15+; 63.3%; alcohol use	PTB; no; yes	Ever, current, past tobacco use	Failure	Strong
Azeez et al^120^ (2019)	2013—2015	South Africa, AFR	Retrospective cohort	910	Adults; > 58.0%; alcohol and drug use, HIV	PTB; yes; no	Current tobacco use	On-treatment mortality	Strong
Castro et al^121^ (2019)	2016	Brazil, AMR	Cross-sectional	180	All ages; 75.6%; alcohol and drug use, 100% HIV	PTB and EPTB; yes (2.9%); no	Current tobacco use	TB mortality, default	Weak
Gupta et al^122^ (2019)	2017—2018	India, SEAR	Prospective cohort	72	18-80; 52.8%; alcohol use, chronic disease	PTB and EPTB; no; no	Ever tobacco use (cigarette, bidi) and smokeless	Unsuccessful	Moderate
Gupte et al^123^ (2019) Thomas et al^124^ (2019)	2014—2017	India, SEAR	Prospective cohort	455	18+; 65.0%; alcohol use, HIV, depression	PTB; no; no	Current, past tobacco use (cigarette, bidi)	Unsuccessful, all-cause mortality, failure, recurrence or relapse	Moderate
Hameed et al^125^ (2019)	2018—2019	Pakistan, EMR	Cross-sectional	170	13-80; 54.1%; HIV, chronic disease	PTB; no; yes	Current tobacco use	On-treatment mortality	Moderate
Ma et al^126^ (2019)	2008—2011	China, WPR	Retrospective cohort	1,256	15+; 72.7%; alcohol use	PTB; no; no	Current, past tobacco use	Unsuccessful, on-treatment mortality, failure, delayed conversion, severity	Moderate
Mathur et al^127^ (2019)	2016—2018	India, SEAR	Prospective cohort	187	All ages; 59.9%; alcohol and drug use, HIV	PTB; NR; no	Current tobacco use	Recurrence or relapse	NA
Nakao et al^128^ (2019)	2008—2016	Japan, WPR	Retrospective cohort	137	All ages; 60.5%; chronic disease	PTB; yes (5.8%); NR	Ever tobacco use	Severity	Moderate
Paunikar et al^129^ (2019)	2015	India, SEAR	Retrospective cohort	440	NR; 56.6%; alcohol and drug use, HIV	PTB and EPTB; NR; yes	Current tobacco use	Default	Moderate
Reimann et al^130^ (2019)	2012—2017	Germany, EUR	Retrospective cohort	247	All ages; 71.3%; alcohol and drug use, HIV, chronic disease	PTB; yes; NR	Ever tobacco use	Delayed conversion, Severity	Strong
Sharma et al^131^ (2019)	2015—2016	India, SEAR	Case-control	741	18+; 60.0%; alcohol and drug use, HIV, DM	PTB; NA; yes	Current tobacco use and smokeless	Drug resistance	Strong
Wardani and Wahono^132^ (2019)	2016	Indonesia, WPR	Case-control	93	All ages; 50.0%; DM	PTB; NR; NR	Current tobacco use	Delayed conversion	Strong
Ajema et al^133^ (2020)	2017	Ethiopia, AFR	Cross-sectional	249	15+ years; alcohol and drug use, HIV	PTB and EPTB; NR; yes	Current tobacco use	Nonadherence	Moderate
Bezerra et al^134^ (2020)	2012—2019	Brazil, AMR	Prospective cohort	148	18+; 65.0%; alcohol and drug use, HIV	PTB and EPTB; NR; yes	Ever tobacco use	Default	Strong
Khan et al^135^ (2020)	2006—2009	Malaysia, WPR	Retrospective cohort	9,337	All ages; 69.0%; alcohol and drug use, chronic disease	PTB and EPTB; NR; yes	Ever tobacco use	On-treatment mortality, default, nonadherence	Strong
Pore et al^136^ (2020)	2016—2017	India, SEAR	Cross-sectional	88	18-70; 77.3%; alcohol use	NR; yes (1.1%); yes	Current tobacco use and smokeless	Nonadherence	NA
Sembiah et al^137^ (2020)	2014—2017	India, SEAR	Prospective cohort	662	18+; 53.2%; alcohol use, DM	PTB and EPTB; NR; yes	Current tobacco use	Unsuccessful	NA
Serpoosh et al^138^ (2020)	2010—2018	Iran, EMR	Case-control	286	All ages; > 50.0%; drug use	NR	Current tobacco use	Failure	Moderate
Takasaka et al^139^ (2020)	2015—2018	Japan, WPR	Retrospective cohort	79	40+; 100%; alcohol use, chronic disease	PTB; no; NR	Ever tobacco use	Delayed conversion	Moderate
Tok et al^140^ (2020)	2014—2017	Malaysia, WPR	Retrospective cohort	97,505	All ages; 64.3%; HIV	PTB and EPTB; no; yes	Current tobacco use	Unsuccessful, all-cause mortality	Strong
Asemahagn^141^ (2021)	2019	Ethiopia, AFR	Prospective cohort	282	15+; 59.0%; alcohol use, HIV, DM	PTB; NR; yes	Current tobacco use	Delayed conversion	Strong
Bhatti et al^142^ (2021)	2016—2018	Malaysia, WPR	Retrospective cohort	606	18+; 73.4%; HIV, chronic disease	PTB; NR; yes	Ever, current, past tobacco use	Delayed conversion	Strong
Cao et al^143^ (2021)	2018—2019	China, WPR	Case-control	1,206	14+; 65.2%; alcohol and drug use, chronic disease	PTB; NR; yes	Current tobacco use	Severity	Strong
Carter et al^144^ (2021)	2015—2017	Liberia, AFR	Retrospective cohort	337	14+; 76.3%; alcohol use, HV, cancer	PTB and EPTB; yes (38.3%); yes (19.0%)	Current, past tobacco use	All-cause mortality	Strong
de Vargas et al^145^ (2021)	2018	Brazil, AMR	Prospective cohort	92	18+; 57.6%; alcohol and drug use, HIV	PTB; NR; NR	Current tobacco use	Unsuccessful	Moderate
Kassim et al^146^ (2021)	2016—2017	Somalia, AFR	Cross-sectional	400	15+; 65.5%; HIV, DM	PTB and EPTB; NR; yes	Current tobacco use	Unsuccessful	Moderate
Lin et al^147^ (2021)	2010—2018	China, WPR	Prospective cohort	634	14+; 69.9%; NR	PTB and EPTB; NR; no	Current, past tobacco use	Recurrence or relapse	Strong
Mokti et al^148^ (2021)	2013—2018	Malaysia, WPR	Retrospective cohort	2,641	All ages; 60.2%; HIV, DM	PTB; no; yes	Current tobacco use	Delayed conversion	Strong

AFR = African region; AMR = American region; DM = diabetes mellitus; DRC = Democratic Republic of Congo; DRTB = drug-resistant TB; EMR = Eastern Mediterranean region; EPTB = extrapulmonary TB; EUR = European region; NA = not applicable; NR = not reported; PTB = pulmonary TB; SEAR = South-East Asian region; WPR = Western Pacific region.

The studies were published from 1998 through 2021, covering data from 1989 and with regular publications from 2005 onward. Studies were from all World Health Organization regions: Western Pacific, n = 33, South-East Asian, n = 24; Eastern Mediterranean, n = 20; African, n = 17; European, n = 18; and the Americas, n = 13. The study designs included 91 cohort, 25 case-control, and nine cross-sectional studies, with wide variations in sample size ranging from 52 to > 1 million research participants.

The participants comprised different age groups, with “all ages” included in 21 studies. Most studies (n = 90) included participants older than 15 years, whereas information on age was not reported in three studies. The proportion of male participants was higher than that of female participants in most studies. Eighty-three studies included individuals who used alcohol in their sample, and a smaller number (n = 47) also reported drug use. Among studies that reported comorbidities, HIV, diabetes, and kidney and liver diseases were the most common. Three studies excluded people with HIV, whereas five excluded participants with any comorbidities. The type of TB was not specified in six studies, both pulmonary and extrapulmonary presentations were covered in 50 studies, whereas the remaining studies were limited to just those with pulmonary TB. A total of 41 studies reported a mix of drug-susceptible TB and DRTB, and 46 studies reported a mix of new and retreatment presentations.

Regarding tobacco exposure, 27 studies reported ever tobacco use (vs never tobacco use), 99 studies reported current tobacco use (vs currently not using tobacco), and 19 studies reported former tobacco use (vs never tobacco use). Of these, eight studies specified bidi use in addition to cigarettes and one study mentioned the inclusion of all forms of tobacco use. Eight studies also reported ST use: two on ever use (vs never use) and six on current use (vs no current use). Regarding treatment outcomes, we found recurrence or relapse (n = 20), mortality during treatment (n = 16), all-cause mortality (n = 11), TB mortality (n = 11), default (n = 27), failure (n = 12), unsuccessful treatment (combined mortality, default, and failure; n = 28), delayed sputum conversion (n = 25), treatment nonadherence (n = 8), disease severity (n = 5), and drug resistance development (n = 1). The overall rating on the risk-of-bias assessments was strong for 68 studies, moderate for 41 studies, and weak for five studies (e-Table 2). Risk of bias was not assessed for the remaining nine studies because we could not extract any results from them.

### TB Recurrence or Relapse

**Figure 2:**
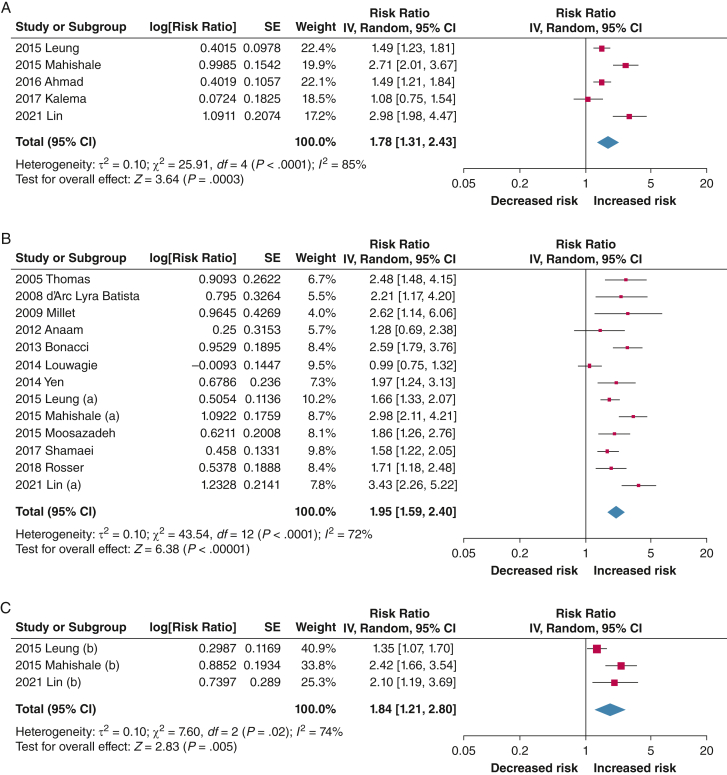
A, Forest plot showing risk of TB recurrence or relapse risk associated with ever using tobacco. B, Forest plot showing risk of TB recurrence or relapse risk associated with current tobacco use. C, Forest plot showing risk of TB recurrence or relapse risk associated with former tobacco use.

Fifteen studies provided the necessary data to be pooled in at least one of the three meta-analyses: five for ever using tobacco,^94^^,^^96^^,^^100^^,^^109^^,^^147^ 13 for current tobacco use,^36^^,^^43^^,^^51^^,^^64^^,^^71^^,^^84^^,^^88^^,^^94^^,^^96^^,^^97^^,^^112^^,^^118^^,^^147^ and three for former tobacco use^94^^,^^96^^,^^147^ (some studies reported on more than one exposure). No studies on ST use were found. Compared with never or no tobacco use, the risk of TB recurrence or relapse was found to be higher with ever tobacco use (pooled RR, 1.78; 95% CI, 1.31-2.43; *I*^2^ = 85%), current tobacco use (RR, 1.95; 95% CI, 1.59-2.40; *I*^2^ = 72%), and former tobacco use (RR, 1.84; 95% CI, 1.21-2.80; *I*^2^ = 74%). All three associations were statistically significant and showed a high degree of heterogeneity (Fig 2A-C). Subgroup analyses showed that variations in study design, quality, and presence of comorbidities could explain some of the heterogeneity, although substantial unexplained heterogeneity within each of these subgroups remained (Table 3); removing the studies that included people with DRTB and retreatment TB did not change the overall findings (e-Fig 1A-M). Funnel plots appeared generally symmetrical, suggesting minimal publication bias (e-Fig 2A-C). The GRADE assessments for all three meta-analyses were very low (e-Fig 3A-C).

**Table 3:** Subgroup Analyses Results

Subgroup	No. of Studies	RR (95% CI)	*I*^2^	Test for Subgroup Differences	
				χ^2^	*P* Value
TB recurrence or relapse					
Ever using tobacco					
Study design				3.40	.07
Prospective	3	2.25 (1.39-3.62)	88%		
Retrospective	2	1.31 (0.96-1.80)	59%		
Study quality				6.19	.01
Strong	4	1.99 (1.43-2.77)	85%		
Weak	1	1.08 (0.75-1.54)	NA		
Comorbidities				3.45	.06
Yes	2	1.31 (0.96-1.80)	60%		
No	2	2.25 (1.40-3.60)	87%		
Current tobacco use					
Study design				21.09	< .01
Prospective	6	2.46 (1.88-3.21)	65%		
Retrospective	6	1.71 (1.45-2.01)	0%		
Cross-sectional	1	0.99 (0.75-1.32)	NA		
Study quality				5.41	.02
Strong	9	2.22 (1.84-2.68)	52%		
Weak	4	1.38 (0.97-1.96)	64%		
Comorbidities				13.48	< .01
Yes	10	1.70 (1.41-2.05)	59%		
No	3	3.00 (2.37-3.80)	0%		
Former tobacco use					
Comorbidities				7.42	.01
Yes	1	1.35 (1.07-1.70)	NA		
No	2	2.32 (1.69-2.80)	0%		
Mortality during treatdsment (current tobacco use)					
Study design				9.87	.01
Prospective	2	1.30 (0.88-1.92)	0%		
Retrospective	6	1.36 (0.94-1.95)	89%		
Cross-sectional	1	5.33 (2.34-12.2)	NA		
Study quality				0.50	.48
Strong	5	1.38 (0.90-2.12)	91%		
Moderate	4	1.77 (1.03-3.03)	38%		
Comorbidities				0.49	.49
Yes	8	1.56 (1.07-2.27)	88%		
No	1	1.29 (0.89-1.87)	NA		

NA = not applicable; RR = risk ratio.

### Mortality During TB Treatment

**Figure 3:**
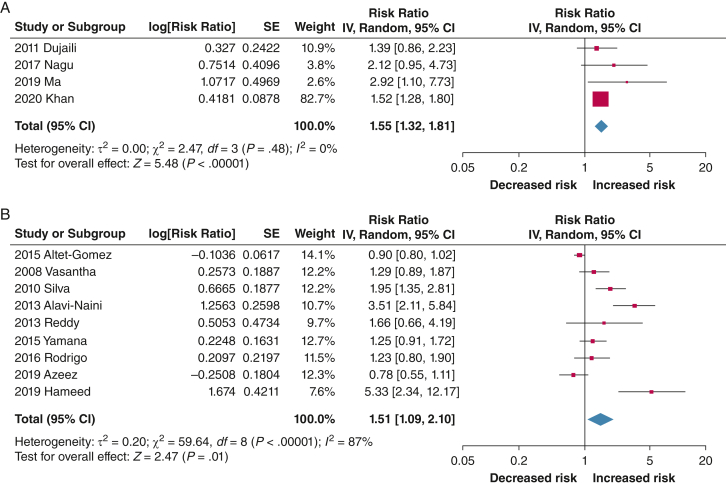
A, Forest plot showing risk of mortality during treatment associated with ever using tobacco among people with TB. B, Forest plot showing risk of mortality during treatment associated with current tobacco use among people with TB.

Of the 13 studies, four provided estimates for ever using tobacco vs never using tobacco,^57^^,^^111^^,^^126^^,^^135^ and nine provided estimates for current tobacco use vs no tobacco use^32^^,^^46^^,^^54^^,^^70^^,^^74^^,^^99^^,^^103^^,^^120^^,^^125^ (Fig 3A, 3B); no estimates were found for former tobacco use or for ST use. Compared with never or no tobacco use, we found increased risk of mortality during treatment associated with ever using tobacco (RR, 1.55; 95% CI, 1.32-1.81; *I*^2^ = 0%) and current tobacco use (RR, 1.51; 95% CI, 1.09-2.10; *I*^2^ = 87%). Only the current tobacco use analysis showed a high degree of heterogeneity, which largely was explained by differences in study design (Table 3). Like recurrence or relapse, removing the studies that included people with DRTB and retreatment TB did not change the overall findings (e-Fig 4A-G). Some funnel plot asymmetry was observed (e-Fig 5A, 5B), and the GRADE assessment was low for both meta-analyses (e-Fig 6A-6B).

### Secondary Outcomes

In addition to mortality during treatment, we included 11 studies on all-cause mortality among people with TB^37^^,^^42^^,^^58^^,^^67^^,^^68^^,^^94^^,^^95^^,^^105^^,^^123^^,^^140^^,^^144^ and 11 on TB mortality.^22^^,^^23^^,^^27^^,^^30^^,^^35^^,^^44^^,^^47^^,^^48^^,^^75^^,^^115^^,^^121^ For all-cause mortality, except for two studies that did not provide risk estimates^58^^,^^67^ and two studies that found no association with current tobacco use,^94^^,^^105^ the remaining study reported increased risk with tobacco use compared with no tobacco use. For TB mortality, all studies reported increased risk with tobacco use and one study report increased risk with ST use in addition (e-Table 3).^35^

Twenty-four of 27 studies on default provided risk estimates for tobacco use.^26^^,^^28^^,^^29^^,^^34^^,^^41^^,^^42^^,^^45^^,^^50^^,^^56^, ^57^, ^58^, ^59^^,^^62^^,^^67^^,^^74^^,^^76^^,^^79^^,^^80^^,^^82^^,^^90^^,^^94^^,^^98^^,^^102^^,^^121^^,^^129^^,^^134^^,^^135^ Except for one study,^62^ all others reported increased risk with tobacco use compared with never or no tobacco use. Regarding treatment failure, nine of 12 identified studies provided risk estimates, all reporting increased risk with tobacco use.^26^^,^^42^^,^^52^^,^^57^^,^^62^^,^^74^^,^^82^^,^^93^^,^^119^^,^^123^^,^^126^^,^^138^ So-called unsuccessful treatment, which combined any outcome other than cure or completion of treatment, was reported in 28 studies.^24^^,^^49^^,^^60^^,^^61^^,^^65^^,^^68^^,^^71^^,^^73^^,^^78^^,^^80^^,^^85^^,^^87^^,^^90^^,^^91^^,^^94^^,^^95^^,^^104^^,^^107^^,^^108^^,^^114^^,^^117^^,^^122^^,^^123^^,^^126^^,^^137^^,^^140^^,^^145^^,^^146^ Of these, the risk could not be extracted from four studies,^24^^,^^49^^,^^61^^,^^137^ whereas most of the remaining ones reported increased risk, including three studies that included the use of ST products.^91^^,^^104^^,^^122^

An association between tobacco use and delayed sputum conversion was reported in 25 studies,^24^^,^^31^^,^^38^^,^^40^^,^^52^^,^^53^^,^^66^^,^^69^^,^^72^^,^^81^^,^^86^^,^^89^^,^^92^^,^^94^^,^^101^^,^^106^^,^^110^^,^^114^^,^^126^^,^^130^^,^^132^^,^^139^^,^^141^^,^^142^^,^^148^ and all but one study^40^ found increased risks. For treatment nonadherence, effect measures were extracted from seven of eight included studies,^33^^,^^77^^,^^82^^,^^113^^,^^116^^,^^133^^,^^135^^,^^136^ all reporting increased risk associated with tobacco use, and one also reporting increased risk associated with ST use.^77^ Disease severity was indicated by risk of hospitalization or cavitation in five included studies,^32^^,^^126^^,^^128^^,^^130^^,^^143^ and all reported increased risk with tobacco use. Finally, one case-control study reported an increased risk of drug resistance developing with tobacco use compared with no tobacco use.^131^

## Discussion

This systematic review identified a substantial number of epidemiologic studies on the association between tobacco use and TB treatment outcomes, and the synthesis clearly showed an increased risk with tobacco use. For the primary outcomes, tobacco use significantly increased the risk of TB recurrence or relapse and mortality during treatment. To our knowledge, the link between tobacco use and TB recurrence or relapse has not been reviewed systematically since 2007,^12^ and no meta-analysis has been conducted until now, although the need for it has been highlighted.^149^^,^^150^ For mortality, previous reviews largely identified TB mortality estimates,^10^, ^11^, ^12^ which identified the association between tobacco use and TB occurrence, rather than treatment outcomes. A 2010 publication summarized the three 2007 reviews and found them to be consistent on TB mortality,^151^ as did the 2014 US Surgeon General's report.^152^ Although we included the TB mortality studies and reached similar conclusions, our meta-analysis focused on mortality during treatment, because this provided a more objective indication of mortality as a treatment outcome.

Our review also found increased risks for default, failure, nonadherence, and delayed sputum conversion. Most of these outcomes were covered in two recent meta-analyses,^13^^,^^14^ both reporting adverse associations with tobacco use. Although our updated searches identified newer studies, we predicted that further meta-analyses would not change the results. Disease severity and development of DRTB were two additional outcomes we included. However, no meta-analyses were conducted because the definition of severity varied across studies, although only one study reported on development of DRTB. Nonetheless, increased risk with tobacco use was found for risk of hospitalization, risk of cavitation, and risk of drug resistance developing.

To our knowledge, the association between ST and TB treatment outcomes has not been reviewed previously. We found eight studies covering unsuccessful treatment, TB mortality, and nonadherence, but the ST-related risks were reported only in six studies. Nonetheless, all but one study found increased risks associated with ST use. Although links between nasal ST (eg, snuff) and increased susceptibility to pulmonary infections have been discussed through mechanisms like decreased mucociliary clearance^153^ and altered microbiome,^154^ further research to elucidate our findings with other ST products are needed. Similarly, among the tobacco use studies, only a few specified bidi and other noncigarette forms, whereas none reported separate effect measures associated with their use.

The key strengths of this review are its rigorous methodologies, the high quality of included studies, and the use of GRADE for the primary outcomes. The limitations, nonetheless, are as follows. First, because the primary studies presented varied estimates (eg, ORs, hazard ratios, and so forth), we used their numbers to calculate RRs for pooling. This meant that studies that did not report the necessary numbers were left out of the meta-analyses. However, these were few and largely reported increased risks with tobacco use. Only two studies on mortality during treatment reported hazard ratios of < 1.00, but one article did not describe the study in adequate detail,^55^ whereas the effect was not statistically significant in the second study.^66^ Our analytical strategy also meant that the effect of important confounders such as age, alcohol, and so forth were not accounted for adequately. The way data were reported on covariates did not allow for their use in metaregression, as originally planned. However, where available within primary studies, we reported adjusted estimates (e-Table 3). Also, when assessing quality, we considered the extent to which studies adjusted for potential confounders.

Another limitation of the meta-analyses is the high heterogeneity: only the ever using tobacco and mortality during treatment analysis showed no heterogeneity. Further, we included studies with combined drug-susceptible TB and DRTB, as well as new and retreatment TB samples. However, we did our best to explain our findings using subgroup and sensitivity analyses. We found that differences in study design, quality, and participant characteristics explained some of the heterogeneity and that removing the studies that included people with DRTB and retreatment TB did not change the overall findings. Additional sources of heterogeneity likely included the geographical spread of studies and the different tobacco products used, but not enough information was available for further exploration. We noted some funnel plot asymmetry in the mortality analyses, suggesting the possibility of publication bias. However, this also may be the result of heterogeneity and chance^155^ and was not assessed further. We also could not rule out the possibility of bias from five studies that were excluded because of language restrictions. Finally, the GRADE assessments for all meta-analyses were either very low or low, suggesting that the true effect may differ from our estimates. However, we believe this was explained largely by the observational study designs and the lack of dose-response effects in most included studies.

## Interpretation

Taken together, our findings show increased risk of TB recurrence or relapse and mortality during treatment with tobacco use compared with never or no tobacco use. Tobacco use is also a clear risk factor for other unfavorable TB treatment outcomes, as documented in earlier reviews. Although evidence is limited on ST, it still suggests that we need to be cognizant of the risks associated with its use, especially given its disproportionately high prevalence in LMICs.^156^ The integration of tobacco cessation within TB services offers a viable option, particularly in LMICs.^12^ A large proportion of people with TB who use tobacco are willing to stop, and those who stop tobacco use have better treatment success (91% vs 80%; *P* < .001) and lower relapse rates (6% vs 14%; *P* < .001).^157^ The results of our review provide additional evidence to invest in these policies and practices to reduce the global TB and tobacco-related disease burden.

## Funding/Support

The authors have reported to *CHEST* that no funding was received for this study.

## Financial/Nonfinancial Disclosures

None declared.

## Supplementary Data

e-Online Data
