# Supplementary material for: Is Tobacco Use Associated With Risk of Recurrence and Mortality Among People With TB? A Systematic Review and Meta-Analysis
Source: Chest. 2023 Aug 29;165(1):22–47. doi: 10.1016/j.chest.2023.08.021 (PMC10790178; doi:10.1016/j.chest.2023.08.021)
Supplement: e-Online Data [file mmc1.docx]

# TB-T Review Supplement – Table of Contents

[e-Appendix 1: PRISMA Checklist 3](#_Toc137703346)

[e-Appendix 2: Database searches 5](#_Toc137703347)

[Database: Ovid MEDLINE(R) 1946 to 2016 December 09 5](#_Toc137703348)

[Database: EMBASE 1974 to 2016 December 09 5](#_Toc137703349)

[Database: CINAHL 2016 December 09 6](#_Toc137703350)

[e-Appendix 3: List of excluded studies (n=116, near-misses=26) 8](#_Toc137703351)

[e-Table 1: Numbers used in meta-analysis for primary review outcomes 11](#_Toc137703352)

[e-Table 2: Quality assessment using EPPHP Assessment Tool 12](#_Toc137703353)

[e-Table 3: Unadjusted and adjusted effect measures reported in included studies 14](#_Toc137703354)

[e-Figure 1: Forest plots of subgroup and sensitivity analyses for TB recurrence/relapse 20](#_Toc137703355)

[a. Ever-smoking – subgroup, study design 20](#_Toc137703356)

[b. Ever-smoking – subgroup, study quality 20](#_Toc137703357)

[c. Ever-smoking – subgroup, comorbidities 21](#_Toc137703358)

[d. Ever-smoking – sensitivity, removing DR-TB studies 21](#_Toc137703359)

[e. Ever-smoking – sensitivity, removing retreatment-TB studies 21](#_Toc137703360)

[f. Current-smoking – subgroup, study design 22](#_Toc137703361)

[g. Current-smoking – subgroup, study quality 22](#_Toc137703362)

[h. Current-smoking – subgroup, comorbidities 23](#_Toc137703363)

[i. Current-smoking – sensitivity, removing DR-TB studies 23](#_Toc137703364)

[j. Current-smoking – sensitivity, removing retreatment-TB studies 24](#_Toc137703365)

[k. Past-smoking – subgroup, comorbidities 24](#_Toc137703366)

[l. Past-smoking – sensitivity, removing DR-TB studies 24](#_Toc137703367)

[m. Past-smoking – sensitivity, removing retreatment-TB studies 24](#_Toc137703368)

[e-Figure 2: Publication bias for TB recurrence/relapse 25](#_Toc137703369)

[a. Ever-smoking – funnel plot 25](#_Toc137703370)

[b. Current-smoking – funnel plot 25](#_Toc137703371)

[c. Past-smoking – funnel plot 26](#_Toc137703372)

[e-Figure 3: GRADE for TB recurrence/relapse 26](#_Toc137703373)

[a. Ever-smoking 26](#_Toc137703374)

[b. Current-smoking 27](#_Toc137703375)

[c. Past-smoking 27](#_Toc137703376)

[e-Figure 4: Forest plots of subgroup and sensitivity analyses for Mortality during TB treatment 28](#_Toc137703377)

[a. Ever-smoking – sensitivity, removing DR-TB studies (no studies removed) 28](#_Toc137703378)

[b. Ever-smoking – sensitivity, removing retreatment-TB studies 28](#_Toc137703379)

[c. Current-smoking – subgroup, study design 29](#_Toc137703380)

[d. Current-smoking – subgroup, study quality 30](#_Toc137703381)

[e. Current-smoking – subgroup, comorbidities 31](#_Toc137703382)

[f. Current-smoking – sensitivity, removing DR-TB studies 31](#_Toc137703383)

[g. Current-smoking – sensitivity, removing retreatment-TB studies 32](#_Toc137703384)

[e-Figure 5: Publication bias for Mortality during TB treatment 32](#_Toc137703385)

[a. Ever-smoking – funnel plot 32](#_Toc137703386)

[b. Current-smoking – funnel plot 33](#_Toc137703387)

[e-Figure 6: GRADE for Mortality during TB treatment 33](#_Toc137703388)

[a. Ever-smoking 33](#_Toc137703389)

[b. Current-smoking 34](#_Toc137703390)

#

# e-Appendix 1: PRISMA Checklist

| **Section and Topic** | **Item #** | **Checklist item** | **Location where item is reported** |
| --- | --- | --- | --- |
| **TITLE** | | |  |
| Title | 1 | Identify the report as a systematic review. | Pg. 1 |
| **ABSTRACT** | | |  |
| Abstract | 2 | See the PRISMA 2020 for Abstracts checklist. | Pg. 3 |
| **INTRODUCTION** | | |  |
| Rationale | 3 | Describe the rationale for the review in the context of existing knowledge. | Pg. 4 |
| Objectives | 4 | Provide an explicit statement of the objective(s) or question(s) the review addresses. | Pg. 4 |
| **METHODS** | | |  |
| Eligibility criteria | 5 | Specify the inclusion and exclusion criteria for the review and how studies were grouped for the syntheses. | Pg. 5, 6 |
| Information sources | 6 | Specify all databases, registers, websites, organisations, reference lists and other sources searched or consulted to identify studies. Specify the date when each source was last searched or consulted. | Pg. 5 |
| Search strategy | 7 | Present the full search strategies for all databases, registers and websites, including any filters and limits used. | Appendix 2 |
| Selection process | 8 | Specify the methods used to decide whether a study met the inclusion criteria of the review, including how many reviewers screened each record and each report retrieved, whether they worked independently, and if applicable, details of automation tools used in the process. | Pg. 5, 6 |
| Data collection process | 9 | Specify the methods used to collect data from reports, including how many reviewers collected data from each report, whether they worked independently, any processes for obtaining or confirming data from study investigators, and if applicable, details of automation tools used in the process. | Pg. 5, 6 |
| Data items | 10a | List and define all outcomes for which data were sought. Specify whether all results that were compatible with each outcome domain in each study were sought (e.g. for all measures, time points, analyses), and if not, the methods used to decide which results to collect. | Pg. 5, 6 |
|  | 10b | List and define all other variables for which data were sought (e.g. participant and intervention characteristics, funding sources). Describe any assumptions made about any missing or unclear information. | Pg. 5, 6 |
| Study risk of bias assessment | 11 | Specify the methods used to assess risk of bias in the included studies, including details of the tool(s) used, how many reviewers assessed each study and whether they worked independently, and if applicable, details of automation tools used in the process. | Pg. 6 |
| Effect measures | 12 | Specify for each outcome the effect measure(s) (e.g. risk ratio, mean difference) used in the synthesis or presentation of results. | Pg. 6 |
| Synthesis methods | 13a | Describe the processes used to decide which studies were eligible for each synthesis (e.g. tabulating the study intervention characteristics and comparing against the planned groups for each synthesis (item #5)). | Pg. 6 |
|  | 13b | Describe any methods required to prepare the data for presentation or synthesis, such as handling of missing summary statistics, or data conversions. | Pg. 6 |
|  | 13c | Describe any methods used to tabulate or visually display results of individual studies and syntheses. | Pg. 6 |
|  | 13d | Describe any methods used to synthesize results and provide a rationale for the choice(s). If meta-analysis was performed, describe the model(s), method(s) to identify the presence and extent of statistical heterogeneity, and software package(s) used. | Pg. 6 |
|  | 13e | Describe any methods used to explore possible causes of heterogeneity among study results (e.g. subgroup analysis, meta-regression). | Pg. 6 |
|  | 13f | Describe any sensitivity analyses conducted to assess robustness of the synthesized results. | Pg. 6 |
| Reporting bias assessment | 14 | Describe any methods used to assess risk of bias due to missing results in a synthesis (arising from reporting biases). | Pg. 6 |
| Certainty assessment | 15 | Describe any methods used to assess certainty (or confidence) in the body of evidence for an outcome. | Pg. 6 |
| **RESULTS** | | |  |
| Study selection | 16a | Describe the results of the search and selection process, from the number of records identified in the search to the number of studies included in the review, ideally using a flow diagram. | Pg. 6, 7, Figure 1 |
|  | 16b | Cite studies that might appear to meet the inclusion criteria, but which were excluded, and explain why they were excluded. | Pg. 6 (also appendix 3) |
| Study characteristics | 17 | Cite each included study and present its characteristics. | Pg. 7, Table 2 (also appendix 5) |
| Risk of bias in studies | 18 | Present assessments of risk of bias for each included study. | Pg. 7, Table 2 (also appendix 4) |
| Results of individual studies | 19 | For all outcomes, present, for each study: (a) summary statistics for each group (where appropriate) and (b) an effect estimate and its precision (e.g. confidence/credible interval), ideally using structured tables or plots. | Pg. 8, 9, Figures 2 and 3 (also appendix 5) |
| Results of syntheses | 20a | For each synthesis, briefly summarise the characteristics and risk of bias among contributing studies. | Pg. 8, 9, Table 3 |
|  | 20b | Present results of all statistical syntheses conducted. If meta-analysis was done, present for each the summary estimate and its precision (e.g. confidence/credible interval) and measures of statistical heterogeneity. If comparing groups, describe the direction of the effect. | Pg. 8, 9 |
|  | 20c | Present results of all investigations of possible causes of heterogeneity among study results. | Pg. 8, Table 3 (also appendix 6) |
|  | 20d | Present results of all sensitivity analyses conducted to assess the robustness of the synthesized results. | Pg. 8 (also appendix 6) |
| Reporting biases | 21 | Present assessments of risk of bias due to missing results (arising from reporting biases) for each synthesis assessed. | Pg. 8 (also appendix 7) |
| Certainty of evidence | 22 | Present assessments of certainty (or confidence) in the body of evidence for each outcome assessed. | Pg. 8 (also appendix8) |
| **DISCUSSION** | | |  |
| Discussion | 23a | Provide a general interpretation of the results in the context of other evidence. | Pg. 9, 10 |
|  | 23b | Discuss any limitations of the evidence included in the review. | Pg. 10 |
|  | 23c | Discuss any limitations of the review processes used. | Pg. 10 |
|  | 23d | Discuss implications of the results for practice, policy, and future research. | Pg. 10, 11 |
| **OTHER INFORMATION** | | |  |
| Registration and protocol | 24a | Provide registration information for the review, including register name and registration number, or state that the review was not registered. | Pg. 5 |
|  | 24b | Indicate where the review protocol can be accessed, or state that a protocol was not prepared. | PROSPERO |
|  | 24c | Describe and explain any amendments to information provided at registration or in the protocol. | NA |
| Support | 25 | Describe sources of financial or non-financial support for the review, and the role of the funders or sponsors in the review. | NA |
| Competing interests | 26 | Declare any competing interests of review authors. | Pg. 22 |
| Availability of data, code and other materials | 27 | Report which of the following are publicly available and where they can be found: template data collection forms; data extracted from included studies; data used for all analyses; analytic code; any other materials used in the review. | Extracted data – Tables and appendices |

#

# e-Appendix 2: Database searches

## Database: Ovid MEDLINE(R) 1946 to 2016 December 09

1 Meta-Analysis as Topic/ (16962)

2 meta analy$.tw. (92746)

3 metaanaly$.tw. (1617)

4 Meta-Analysis/ (81122)

5 or/1-4 (122348)

6 Epidemiologic studies/ (7951)

7 exp case control studies/ (877001)

8 exp cohort studies/ (1714296)

9 Case control.tw. (98682)

10 (cohort adj (study or studies)).tw. (124995)

11 Cohort analy$.tw. (5123)

12 (Follow up adj (study or studies)).tw. (42366)

13 (observational adj (study or studies)).tw. (61169)

14 Longitudinal.tw. (179023)

15 Retrospective.tw. (336602)

16 Cross sectional.tw. (220134)

17 Cross-sectional studies/ (255005)

18 or/5-17 (2455966)

19 exp smokeless/ (525)

20 (Gutka or Naswar or Nas or Mishri or Khiwam or Zarda or Mawa or Pan-masala or

Gudhakhu or Tuibur or Gul or Snuff or Twist or Plug or Quid or Chimo or Iqmik or Loose-leaf or Tobacco-toothpaste or Tobacco-tablets or Toombak or Khaini or Paan).mp. [mp=title, abstract, original title, name of substance word, subject heading word, keyword heading word, protocol supplementary concept word, rare disease supplementary concept word, unique identifier] (25033)

21 19 or 20 (25557)

22 exp tobacco/ (29032)

23 smok*.tw. (230406)

24 cigarette*.tw. (61285)

25 22 or 23 or 24 (256970)

26 (animals not humans).sh. (4636436)

27 (21 and 18) not 26 (3130) [Category 1: smokeless + all design terms]

28 (25 and 18) not 26 (94057) [Category 2: smoking + all design terms]

29 exp tuberculosis/ or tuberculosis.mp. or exp antitubercular drugs/ (265814)

30 27 and 29 (12) [set 1: smokeless + TB]

31 28 and 29 (713) [set 2: smoking + TB]

32 30 or 31 (721) [all tobacco use forms + TB]

## Database: EMBASE 1974 to 2016 December 09

1 exp Meta Analysis/ (154113)

2 ((meta adj analy$) or metaanalys$).tw. (136813)

3 Case control study/ (123393)

4 Longitudinal study/ (106414)

5 Retrospective study/ (519614)

6 Prospective study/ (389765)

7 Randomized controlled trials/ (126341)

8 6 not 7 (384962)

9 Cohort analysis/ (304936)

10 (Cohort adj (study or studies)).mp. (186151)

11 (Case control adj (study or studies)).tw. (101351)

12 (follow up adj (study or studies)).tw. (53815)

13 (observational adj (study or studies)).tw. (102356)

14 (epidemiologic$ adj (study or studies)).tw. (89393)

15 (cross sectional adj (study or studies)).tw. (132711)

16 or/1-5,8-15 (1754306)

17 exp smokeless/ (4217)

18 Gutka/ (8)

19 Naswar/ (1)

20 Nas/ (3)

21 Mishri/ (1)

22 Khiwam/ (0)

23 Zarda/ (187)

24 Snuff/ (4217)

25 Gul/ (0)

26 Tuibur/ (0)

27 Gudhakhu/ (0)

28 Pan-masala/ (22)

29 Mawa/ (3)

30 Paan/ (2)

31 Khaini/ (3)

32 Toombak/ (1)

33 Tobacco-tablets/ (0)

34 Tobacco-toothpaste/ (0)

35 Loose-leaf/ (0)

36 Iqmik/ (0)

37 Chimo/ (1)

38 Quid/ (122)

39 Plug/ (12)

40 Twist/ (5)

41 or/17-40 (4570)

42 exp tobacco/ (54343)

43 smok*.tw. (317604)

44 cigarette*.tw. (72696)

45 exp animals/ not humans.sh. (22769754)

46 42 or 43 or 44 (355348)

47 (41 and 16) not 45 (13) [Category 1: smokeless + all design terms]

48 (46 and 16) not 45 (3664) [Category 2: smoking + all design terms]

49 exp tuberculosis/ or tuberculosis.mp. or exp antitubercular drugs/ (242118)

50 47 and 49 (1) [set 1: smokeless + TB]

51 48 and 49 (26) [set 2: smoking + TB]

52 50 or 51 (26) [all tobacco use forms + TB]

## Database: CINAHL 2016 December 09

S22 S20 or S21 [all tobacco use forms + TB] 38

S21 S18 and S19 [set 2: smoking + TB] 37

S20 S17 and S19 [set 1: smokeless + TB] 3

S19 (MH &quot;Tuberculosis+&quot;) OR (MH &quot;Tuberculosis, Pulmonary&quot;) 16,255

S18 (S15 and S9) not S16 [Category 2: smoking + all design terms] 13,597

S17 (S14 and S9) not S16 [Category 1: smokeless + all design terms] 735

S16 &quot;Animal studies&quot;/ 76,536

S15 S12 or S13 54,356

S14 S10 or S11 4,790

S13 (MH &quot;Smoking+&quot;) 51,859

S12 (MH &quot;Tobacco&quot;) 5,743

S11 Gutka or Naswar or Nas or Mishri or Khiwam or Zarda or Mawa or Pan-masala or Gudhakhu or Tuibur or Gul or Snuff or Twist or Plug or Quid or Chimo or Iqmik or Loose-leaf or Tobacco-toothpaste or Tobacco-tablets or Toombak or Khaini or Paan 3,542

S10 smokeless tobacco 1,446

S9 S1 or S2 or S3 or S4 or S5 or S6 or S7 or S8 479,410

S8 (observational adj (study or studies)).tw. 302

S7 (cohort adj (study or studies)).tw. 305

S6 Cross sectional studies/ 126,549

S5 Nonconcurrent prospective studies/ 192

S4 Correlational studies/ 20,683

S3 Exp case control studies/ 1

S2 Prospective studies/ 316,311

S1 Meta analysis/ 42,291

# e-Appendix 3: List of excluded studies (n=116, near-misses=26)

| **Studies ‘Not retrieved’**   1. 2008 Diallo (not available in any British library) |
| --- |
| **Studies with ‘No eligible outcome/exposure’**   1. 2005 Calpe 2. 2005 Kiwuwa 3. 2005 Ruddy 4. 2007 Cantalice Filho, Boia, and Sant Anna 5. 2009 Basnet 6. 2010 Marahatta 7. 2011 Okumura 8. 2011 Qazi 9. 2012 Bam 10. 2012 Dalton 11. 2013 Apostu and Mihaescu 12. 2013 Mehra, NM assessed ‘initial defaulters’ rather than treatment default 13. 2013 Muwonge 14. 2014 Out, NM 15. 2014 Rifat, NM 16. 2014 Shu 17. 2015 Chao 18. 2015 Valenca 19. 2015 Gajalakshmi and Kanimozhi 20. 2015 Marahatta, NM 21. 2016 Zhang, NM 22. 2017 Chio 23. 2017 Cunha, NM 24. 2017 Fregona 25. 2017 Gaifer, NM 26. 2017 Mwiru 27. 2018 Echazarre 28. 2018 Friis 29. 2018 Jin 30. 2018 Kombila, 31. 2018 Pourostad 32. 2018 Stjepanov 33. 2019 Ali 34. 2019 Asemahagn, NM 35. 2019 Elduma 36. 2019 Ganmaa 37. 2019 Hoyt, NM 38. 2019 Kim 39. 2019 Lei 40. 2020 Khosa 41. 2020 Tewatia 42. 2008 Talay 43. 2009 Lin 44. 2016 Belard 45. 2017 Harling, NM 46. 2016 Hernandez-Guerrero 47. 2020 Adegbite 48. 2020 An, NM 49. 2020 Bernier 50. 2020 Eddabra 51. 2020 Gompo 52. 2020 Kumar 53. 2020 Marshall 54. 2020 Patel 55. 2020 Shimouchi 56. 2020 Tiamiyu 57. 2020 Velayutham 58. 2021 Ahmad 59. 2015 Atif, NM |
| **Studies with ‘Not relevant design’**   1. 1990 Howell, Kelly and Clancy 2. 1996 Tan, Sin Fai Lam, and Chew 3. 2009 Tang and Ali 4. 2010 Fekih 5. 2013 Mor 6. 2017 Duro, NM 7. 2017 Mukhtar 8. 2018 Amere 9. 2018 Ben saad 10. 2018 Mjid 11. 2019 Assao, NM 12. 2019 Fafa Cisse 13. 2019 Muller 14. 2017 Masjedi 15. 2020 Burusie 16. 2020 Menon 17. 2020 Obore 18. 2020 Peer 19. 2011 Awaisu |
| **Wrong population**   1. 2014 Duraisamy 2. 2014 Magee, NM included MDR TB patients at entry-point 3. 2015 Li, NM all retreatment cases 4. 2016 Belchior, NM retreatment patients 5. 2017 Moro 6. 2018 Azeez 7. 2019 Naz 8. 2006 Laviagne, NM 9. 2020 Alipour 10. 2020 Burney 11. 2020 Chen, NM 12. 2020 Ketema, NM 13. 2020 Koslik 14. 2020 Kwak, NM 15. 2020 Lee 16. 2020 Lule 17. 2020 Mondal 18. 2020 Reichler 19. 2020 Sambas, NM 20. 2020 Velan 21. 2020 Welekidan, NM 22. 2020 Yamanaka 23. 2021 Baluku, NM 24. 2021 Diktanas 25. 2021 Ilic 26. 2021 Lange 27. 2021 Ping 28. 2011 Dolma, NM, all retreatment patients 29. 2021 Baluku, NM, dr-tb |
| **Studies that were ‘Not translated’**   1. 2001 Albuquerque 2. 2009 Shprykov 3. 2010 Racil 4. 2012 Racil 5. 2018 Kombila, radiological |
| **Other reasons for exclusion**   1. 2014 Ajagbe, Kabir, and O'Connor – retracted paper 2. 2017 Mohd Hanafiah, biomarker study 3. 2020 Khan, diagnostic testing study 4. 2020 Li, gene expression study |

# e-Table 1: Numbers used in meta-analysis for primary review outcomes

| **Study ID** | **Exposed** | **Unexposed** | **Cases in exposed** | **Cases in unexposed** |
| --- | --- | --- | --- | --- |
| **Recurrence/relapse, ever-smoking (n=5)** | | | | |
| 2015 Leung | 6832 | 6517 | 260 | 166 |
| 2015 Mahishale | 757 | 1593 | 89 | 69 |
| 2016 Ahmad | 118 | 214 | 75 | 91 |
| 2017 Kalema | 77 | 157 | 29 | 55 |
| 2021 Lin | 277 | 357 | 67 | 29 |
| **Recurrence/relapse, current-smoking (n=13)** | | | | |
| 2005 Thomas | 226 | 260 | 41 | 19 |
| 2008 d'Arc Lyra Batista | 152 | 553 | 14 | 23 |
| 2009 Millet | 363 | 303 | 22 | 7 |
| 2012 Anaam | 41 | 179 | 10 | 34 |
| 2013 Bonacci | 145 | 752 | 34 | 68 |
| 2014 Louwagie | 409 | 1468 | 53 | 192 |
| 2014 Yen | 985 | 4582 | 25 | 59 |
| 2015 Leung | 3221 | 6517 | 136 | 166 |
| 2015 Mahishale | 395 | 1593 | 51 | 69 |
| 2015 Moosazadeh | 231 | 1040 | 31 | 75 |
| 2017 Shamaei | 145 | 302 | 63 | 83 |
| 2018 Rosser | 39 | 207 | 20 | 62 |
| 2021 Lin | 183 | 357 | 51 | 29 |
| **Recurrence/relapse, past-smoking (n=3)** | | | | |
| 2015 Leung | 3611 | 6517 | 124 | 166 |
| 2015 Mahishale | 362 | 1593 | 38 | 69 |
| 2021 Lin | 94 | 357 | 16 | 29 |
| **Mortality during TB treatment, ever-smoking (n=4)** | | | | |
| 2011 Dujaili | 274 | 250 | 38 | 25 |
| 2017 Nagu (RR reported) | - | - | - | - |
| 2019 Ma | 767 | 560 | 20 | 5 |
| 2020 Khan | 4313 | 5024 | 283 | 217 |
| **Mortality during TB treatment, current-smoking (n=9)** | | | | |
| 2005 Altet-Gomez | 4557 | 8481 | 358 | 739 |
| 2008 Vasantha | 1453 | 2060 | 52 | 57 |
| 2010 Silva | 93 | 261 | 34 | 49 |
| 2013 Alavi-Naini | 339 | 376 | 57 | 18 |
| 2013 Reddy | 201 | 212 | 11 | 7 |
| 2015 Yamana | 283 | 479 | 54 | 73 |
| 2016 Rodrigo | 2640 | 2494 | 47 | 36 |
| 2019 Azeez | 581 | 229 | 77 | 39 |
| 2019 Hameed | 51 | 119 | 16 | 7 |

# e-Table 2: Quality assessment using EPPHP Assessment Tool

| **Study ID** | **Overall**  **(S/M/W)** | **Selection bias (S/M/W)** | **Study design (S/M/W)** | **Confounder (S/M/W)** | **Blinding (S/M/W)** | **Data collection (S/M/W)** | **Withdrawal/dropouts (S/M/W)** |
| --- | --- | --- | --- | --- | --- | --- | --- |
| 1998 Liu | Strong | S | M | M | M | S | M |
| 2001 Lam | Mod | M | M | M | M | S | W |
| 2002 Leung, 2003 Leung | Mod | S | M | W | S | S | S |
| 2002 Santha | Mod | S | M | W | S | S | S |
| 2003 Gajalakshmi | Strong | S | M | S | M | S | M |
| 2003 Salami | Mod | M | M | W | S | S | S |
| 2004 Chang | Strong | S | M | S | S | S | M |
| 2004 Sitas | Strong | S | M | M | M | S | M |
| 2005 Abal | Mod | S | M | W | M | S | M |
| 2005 Altet-Gomez | Strong | S | M | S | S | S | M |
| 2005 Balbay | Strong | M | M | S | M | S | S |
| 2005 Chandrasekaran | Mod | M | M | W | M | M | S |
| 2005 Gupta | Strong | S | M | M | S | S | S |
| 2005 Thomas | Strong | S | M | M | M | S | S |
| 2006 Kolappan | Mod | W | M | S | S | S | M |
| 2007 Babb | Strong | M | M | S | S | S | S |
| 2007 Cacho | Strong | M | M | M | M | S | M |
| 2007 Guler | Weak | W | M | S | S | S | W |
| 2007 Jakubowiak | Mod | M | M | W | S | S | S |
| 2007 Wang | Strong | M | M | S | S | S | S |
| 2008 d'Arc Batista | Strong | M | M | S | M | S | S |
| 2008 Jha | Strong | M | M | M | M | S | M |
| 2008 Pinidiyapathirage | Mod | M | M | W | M | M | M |
| 2008 Vasantha | Mod | W | M | M | M | S | S |
| 2009 Jee | Strong | M | M | M | M | S | M |
| 2009 Jiang | Strong | M | M | M | S | S | M |
| 2009 Kherad | NA | NA | NA | NA | NA | NA | NA |
| 2009 Kittikraisak | Strong | M | M | S | M | S | S |
| 2009 Millet | Mod | S | M | W | M | S | S |
| 2010 Metanat | Weak | W | M | W | M | M | S |
| 2010 Siddiqui | Mod | W | M | M | S | S | M |
| 2010 Silva | Strong | S | M | M | S | S | S |
| 2010 Tabarsi | NA | NA | NA | NA | NA | NA | NA |
| 2010 Vijay | Strong | S | M | S | M | S | M |
| 2011 Dujaili | Strong | S | M | S | M | S | S |
| 2011 Garcia-Garcia | NA | NA | NA | NA | NA | NA | NA |
| 2011 Maruza | Strong | M | M | S | M | S | S |
| 2011 Nik Mahdi | Mod | S | M | W | M | S | M |
| 2011 Solliman | NA | NA | NA | NA | NA | NA | NA |
| 2011 & 2013 Tachfouti | Strong | M | M | S | M | S | M |
| 2012 Anaam | Mod | S | M | W/M | M | S | M |
| 2012 Chiang | Strong | M | M | S | M | S | M |
| 2012 Feng | Mod | M | M | W | S | S | S |
| 2012 Lisha | NA | NA | NA | NA | NA | NA | NA |
| 2012 Tabarsi | Mod | S | M | W | S | S | S |
| 2012 Visser | Strong | M | M | S | S | S | S |
| 2013 Alavi-Naini | Strong | M | M | S | S | S | M |
| 2013 Bonacci | Strong | S | M | S | M | S | S |
| 2013 Maciel | Strong | M | M | M | S | S | M |
| 2013 Mnisi | Mod | M | M | W | S | S | S |
| 2013 Reddy | Mod | M | M | W | M | M | M |
| 2013 Reed | Strong | M | M | S | M | S | M |
| 2013 Slama | Strong | M | M | S | M | S | M |
| 2014 Ahmad | Mod | M | M | W | S | S | S |
| 2014 Alo | Mod | S | M | W | S | S | S |
| 2014 Cherkaoui | Strong | S | M | M | S | S | S |
| 2014 Choi | Mod | M | M | W | M | S | M |
| 2014 de Boer | Strong | M | M | M | S | S | S |
| 2014 & 2015 Ibrahim | Mod | S | W | S | S | S | S |
| 2014 Louwagie | Mod | M | W | S | M | S | M |
| 2014 Lucenko | Mod | S | M | W | S | S | S |
| 2014 Pefura-Yone | Strong | M | M | M | S | S | S |
| 2014 Przybylski | Mod | S | M | W | S | M | S |
| 2014 Yen | Strong | S | M | S | S | S | S |
| 2015 Chuang | Strong | M | M | M | M | S | M |
| 2015 Driessche | Strong | M | M | M | M | S | M |
| 2015 Gegia | Strong | S | M | S | M | S | M |
| 2015 Kanda | Strong | M | M | S | S | S | S |
| 2015 Khan | Mod | M | M | W | M | S | M |
| 2015 Leung | Strong | S | M | S | M | S | M |
| 2015 Liew | Strong | M | M | S | S | S | S |
| 2015 Mahishale | Strong | M | M | M | M | S | S |
| 2015 Moosazadeh | Strong | S | M | S | M | S | M |
| 2015 Roy | Strong | S | M | S | S | S | S |
| 2015 Yamana | Strong | M | M | S | S | S | S |
| 2016 Ahmad | Strong | M | M | M | M | S | M |
| 2016 Ajili | NA | NA | NA | NA | NA | NA | NA |
| 2016 Rathee | Mod | M | M | W | M | S | S |
| 2016 Rodrigo | Mod | M | M | W | M | S | M |
| 2016 Veerakumar | Strong | S | M | S | M | S | S |
| 2016 Yen | Strong | S | M | S | S | S | S |
| 2017 Altet | Strong | M | M | S | M | S | M |
| 2017 Balian | Strong | S | M | S | M | S | S |
| 2017 Jaber | Strong | M | M | S | M | S | M |
| 2017 Kalema | Weak | M | M | W | M | S | W |
| 2017 Musteikiene | Strong | M | M | M | S | S | S |
| 2017 Nagu | Strong | M | M | S | M | S | S |
| 2017 Shamaei | Mod | M | M | S | M | S | W |
| 2017 Tola | Weak | M | W | W | M | S | M |
| 2018 Cailleaux-Cezar | Strong | M | M | S | S | S | M |
| 2018 Dizaji | Mod | M | M | S | M | S | W |
| 2018 Madeira | Strong | M | M | S | M | S | M |
| 2018 Mukhtar | Strong | M | M | S | M | S | S |
| 2018 Rosser | Strong | M | M | S | M | S | M |
| 2019 Aguilar | Strong | M | M | S | M | S | M |
| 2019 Azeez | Strong | M | M | S | M | S | M |
| 2019 Castro | Weak | M | W | W | M | S | M |
| 2019 Gupta | Mod | M | M | W | M | S | S |
| 2019 Gupte & Thomas | Mod | M | M | S | S | S | W |
| 2019 Hameed | Mod | M | M | W | S | S | S |
| 2019 Ma | Mod | S | M | W | S | S | M |
| 2019 Mathur | NA | NA | NA | NA | NA | NA | NA |
| 2019 Nakao | Mod | S | M | W | S | S | S |
| 2019 Paunikar | Mod | S | M | W | S | S | S |
| 2019 Reimann | Strong | M | M | M | S | S | S |
| 2019 Sharma | Strong | M | M | S | S | S | M |
| 2019 Wardani | Strong | M | M | S | S | S | S |
| 2020 Ajema | Mod | S | W | S | M | S | M |
| 2020 Bezerra | Strong | M | M | S | M | S | S |
| 2020 Khan | Strong | M | M | S | S | S | S |
| 2020 Pore | NA | NA | NA | NA | NA | NA | NA |
| 2020 Sembiah | NA | NA | NA | NA | NA | NA | NA |
| 2020 Serpoosh | Mod | M | M | W | S | S | M |
| 2020 Takasaka | Mod | W | M | M | M | S | S |
| 2020 Tok | Strong | S | M | S | S | S | S |
| 2021 Asemahagn | Strong | S | M | S | S | S | S |
| 2021 Bhatti | Strong | S | M | S | S | S | S |
| 2021 Cao | Strong | S | M | S | S | S | S |
| 2021 Carter | Strong | S | M | S | S | S | S |
| 2021 de Vargas | Mod | S | M | W | M | S | S |
| 2021 Kassim | Mod | M | W | S | M | S | M |
| 2021 Lin | Strong | S | M | S | S | S | S |
| 2021 Mokti | Strong | S | M | S | S | S | S |

# e-Table 3: Unadjusted and adjusted effect measures reported in included studies

| **Study** | **Unadjusted effect (95% CI)** | **Adjusted effect (95% CI)** | Confounders |
| --- | --- | --- | --- |
| **OUTCOME 1: Recurrence/relapse (n=20)** | | | |
| 2005 Thomas | OR curr smoke 2.80 (1.50-5.20) | OR curr smoke 3.10 (1.60-6.00) | Alcohol, drug regularity, drug sensitivity |
| 2007 Cacho | NR | OR curr smoke 49.1 (19.8–125.6) | Age, sex, drugs, alcohol, HIV, CXR, AFB |
| 2008 d'Arc Lyra Batista | OR curr smoke 2.33 (1.17–4.66) | OR curr smoke 2.53 (1.23–5.21) | Age, sex, social factors, alcohol, access to health services |
| 2009 Jee | NR | HR curr smoke men 1.20 (1.10-1.30), past men 1.30 (1.20-1.40), curr women 1.00 (0.70-1.40), past women 1.20 (0.90, 1.80) | Age, age-squared, BMI, alcohol |
| 2009 Millet | HR curr smoke 2.60 (1.10-6.20) | NR (not significant) | NA |
| 2012 Anaam | OR curr smoke 1.40 (0.60-3.10) | OR curr >20 vs. 1-20/day 9.40 (1.10-83.90) | Employment, 80% of doses, cavitation, weight, DM |
| 2012 Lisha | NR | NR (Higher risk with smoking) | Age, sex, income, weight, category of treatment, DM, etc. |
| 2013 Bonacci | NR | HR curr light smoke 2.14 (1.18-3.89),  heavy 2.37 (1.12-4.98) | Age, sex, crowding, rural, new patient, HIV, weight, etc. |
| 2014 Louwagie | OR curr smoke 0.99 (0.71-1.37) | OR curr smoke 0.98 (0.51-1.90) | Age, sex, HIV status, ART, household income |
| 2014 Yen | HR curr 1-10/day 1.01(0.37-2.77), >10/day 2.54 (1.54-4.17) | HR curr 1-10/day 0.86 (0.31-2.38), >10/day 2.04 (1.22-3.41) | Age, sex, marital, education, homeless, alcohol, ATT, etc. |
| 2015 Leung | OR curr smoke 2.48 (1.77-3.47), past 1.88 (1.32-2.66) | HR curr smoke 1.63 (1.29–2.06), past 1.33 (1.04–1.71) | Age, sex, ethnicity, residence, employment, alcohol, HIV, etc. |
| 2015 Mahishale | OR curr smoke 3.27 (2.24-4.79), past 2.59 (1.71-3.92) | HR curr smoke 1.68 (1.46-1.98), past 1.43 (1.06-1.61) | Age, duration of smoking, BMI, socioeconomic status, DM |
| 2015 Moosazadeh | OR curr smoke 1.99 (1.28-3.11) | OR curr smoke 2.20 (1.30-3.70) | Age, sex, smear before treatment, smear at 2 months treatment, treatment result, DM |
| 2016 Ahmad | OR ever smoke 2.05 (1.32-3.19) | OR ever smoke 2.20 (1.30-3.70) | Age, sex, comorbidities (by exclusion) |
| 2017 Kalema | OR ever smoke 1.12 (0.64-1.97) | NR | NA |
| 2017 Shamaei | OR curr smoke 2.03 (1.34–3.07) | OR curr smoke 1.91 (1.15-3.18) | Age, sex, marital, nationality, TB type, ATT, comorbidities, etc. |
| 2018 Rosser | OR curr smoke 2.80 (1.40-5.60) | OR curr smoke 3.80 (1.10-13.10) | Age, sex, weight, ethnicity, alcohol, comorbidities, etc. |
| 2019 Gupte, 2019 Thomas | NR | IRR curr smoke 2.94 (1.30–6.67) | Age, sex, family income, HIV, DM, chest x-ray cavity, smear |
| 2019 Mathur | NR | NR | NA |
| 2021 Lin | HR curr smoke 2.18 (1.18-4.04), past 3.79 (2.40-5.98) | HR curr smoke 2.52 (1.38-4.62), past 1.31 (0.62-2.76) | Age, sex, type of TB, lung cavitation |
| OUTCOME 2: Mortality during treatment (n=16) | | | |
| 2005 Altet-Gomez | OR curr smoke 0.89 (0.78-1.02) | NR | Age, sex, alcohol, infection site |
| 2008 Vasantha | OR curr smoke 1.30 (0.89-1.91) | HR curr smoke 0.73 (0.44, 1.23) | Age, sex, treatment category, education, occupation, alcohol, etc. |
| 2010 Silva | OR curr smoke 2.02 (1.02–3.98) | OR curr smoke 2.14 (1.07–4.28) | Duration of hospitalization, schooling, HIV, comorbidity, etc. |
| 2010 Tabarsi | HR curr smoke 0.54 (no CI) | HR curr smoke 0.62 (0.44-0.87) | Age, TB history, comorbidity, involvement of lung, cavity, etc. |
| 2011 Dujaili | OR ever smoke 1.45 (0.84-2.47) | OR ever smoke 0.96 (0.48-1.92) | Age, sex, alcohol use, IVDU, history of chronic disease |
| 2012 Feng | HR curr smoke 0.78 (0.57–1.08) | NR | NA |
| 2013 Alavi-Naini | OR curr smoke 4.00 (2.30-7.00) | OR curr smoke 12.90 (3.90-27.30) | Drug use, hepatitis, DM, previous TB, positive sputum smear, etc. |
| 2013 Reddy | OR curr smoke 1.69 (0.64-4.46) | NR | NA |
| 2015 Driessche | HR ever smoke 2.00 (1.10 – 3.63) | HR ever smoke 0.87 (0.38 – 1.98) | Age, sex, weight, CD4 count, ART, EPTB, alcohol, etc. |
| 2015 Yamana | OR curr <=50 PY 1.19 (0.77-1.85), >50 1.64 (0.92-2.94) | OR curr <=50 PY 1.52 (0.89-2.57), >50 2.10 (1.08-4.08) | Age, sex, BMI, urgency of admission, comorbidities, etc. |
| 2016 Rodrigo | HR curr smoke 1.24 (0.80-1.91) | NR | NA |
| 2017 Nagu | RR ever smoke 2.12 (0.95–4.74) | RR ever smoke 4.89 (1.44–16.55) | Age, sex, HIV, HT, cavitation, BMI, education, duration, etc. |
| 2019 Azeez | NR | OR curr smoke 1.32 (1.77-5.76) | Age, weight, alcohol, diabetes, HIV, CD4 cell count, pregnancy |
| 2019 Hameed | OR curr smoke 7.31 (2.78-19.21) | NR | NA |
| 2019 Ma | OR curr smoke 2.62 (0.50-13.73), past 2.13 (0.67-6.77) | NR | NA |
| 2020 Khan | OR ever smoke 1.55 (1.29 - 1.86) | OR ever smoke 1.57 (1.31 - 1.89) | Age, sex, race, area, employment, alcohol, comorbidity, etc. |
| OUTCOME 3: All-cause mortality (n=11) | | | |
| 2006 Kolappan | NR | HR current smoke 1.1 (0.50-2.30) | Age, treatment outcomes, behavioural factors |
| 2007 Wang | HR ever smoke 1.62 (1.05–2.50) | HR ever smoke 1.42 (0.87–2.31) | Age, sex, underlying disease, symptoms, DR-TB, etc. |
| 2011 Garcia-Garcia | NR | NR | NA |
| 2012 Lisha | NR | NR (higher risk with smoking) | NA |
| 2012 Tabarsi | OR curr smoke 0.11 (0.01-1.24) | NR | NA |
| 2015 Leung | OR current smoke 0.99 (0.83-1.20), past 3.18 (2.77-3.65) | NR | NA |
| 2015 Liew | OR curr smoke 1.40 (1.28–1.54) | OR curr smoke 0.85 (0.74–0.99) | Age, sex, education, type of TB, BCG, DM, HIV, etc. |
| 2016 Yen | NR | OR curr smoke 0.94 (0.59-1.51), past 1.05 (0.67-1.64) | Age, sex, BMI, marital status, education, alcohol, DM, history of TB disease, AFB-smear positivity, etc. |
| 2019 Gupte, 2019 Thomas | NR | IRR current smoke 2.59 (1.29-5.18), Past 2.63 (1.11-6.24) | Age, sex, family income, HIV, DM, chest x-ray cavity, smear |
| 2020 Tok | OR curr smoke 1.25 (1.20-1.31) | OR curr smoke 0.97 (0.92-1.02) | Age, sex, nationality, rural, education, BCG, DM, HIV, etc. |
| 2021 Carter | NR | HR current smoke 3.55 (1.19-10.52), past 3.77 (1.60-8.89) | Age, sex, marital status, occupation, overcrowding, DR-TB |
| **OUTCOME 4: TB mortality (n=11)** | | | |
| 1998 Liu | NR | RR curr smoke men 1.20 (1.12-1.27), women 1.29 (1.13-1.45) | Age |
| 2001 Lam | NR | RR curr smoke men 2.54 (1.24-5.22), women 1.49 (0.18-12.57) | Age, education |
| 2003 Gajalakshmi | NR | RR ever smoke urban 4.50 (4.00-5.00), rural 4.20 (3.70-4.80) | Age, education, SLT |
| 2004 Sitas | NR | OR ever smoke 1.61, (1.23-2.11) | Age, sex, population group, education |
| 2005 Gupta | NR | RR ever smoke men 2.30 (1.68–3.15), SLT 1.46 (1.07–2.00), women smoke 5.92 (2.31–15.17), SLT 1.40 (0.99–2.00) | Age, education |
| 2008 Jha | NR | RR curr smoke men 2.30 (2.10–2.60), women 3.00 (2.40–3.90) | Age, education, alcohol |
| 2009 Jee | NR | HR curr smoke men 1.21 (0.96-1.51), past men 1.37 (1.07-1.75), curr women 1.08 (0.67-1.74), past women 1.98 (1.21-3.24) | Age, BMI, alcohol |
| 2009 Jiang | OR ever smoke men 1.48 (1.40-1.56), women 1.55 (1.43-1.67) | OR ever smoke men 1.50 (1.40-1.60), women 1.60 (1.50-1.70) | Age, locality |
| 2013 Reed | HR curr smoke 1.10 (0.38-3.18) | HR curr smoke 2.10 (0.53-8.30) | Age, sex, education, alcohol, DM, retreatment cases |
| 2018 Dizaji | RR curr smoke 2.50 (1.40-4.20) | RR curr smoke 3.30 (1.20-9.40) | Age, sex, marital status, education, DM, HIV, etc. |
| 2019 Castro | OR curr smoke 1.73 (0.37-8.06) | NR | NA |
| **OUTCOME 5: Default (n=27)** | | | |
| 2002 Santha | OR curr smoke 2.10 (1.30-3.40) | NR | NA |
| 2003 Salami | OR curr smoke 1.61 (1.31-1.98) | NR | NA |
| 2004 Chang | OR ever smoke 2.90 (1.73-4.85) | OR curr 3.00 (1.41-6.39), past 1.84 (0.71-4.75) | Age, sex, residence, alcohol, chronic disease, etc. |
| 2005 Chandrasekaran | OR curr smoke 2.70 (1.80-4.20) | NR (not significant) | NR |
| 2007 Jakubowiak | OR curr smoke 6.37 (2.30-17.70) | NR | NA |
| 2007 Wang | OR ever smoke 1.98 (1.12-3.51) | OR ever smoke 2.45 (1.22-4.93) | Age, sex, underlying disease, duration of symptoms, etc. |
| 2008 Pinidiyapathirage | OR curr (reg) smoke 3.05 (2.19-4.24) | OR curr smoke 1.86 (1.27-2.74) | Type of patient, chest X-ray involvement >3 zones, occupation |
| 2009 Kittikraisak | NR | OR curr smoke 2.50 (1.40-4.50) | Age, incarceration, socioeconomic status, symptoms, etc. |
| 2010 Vijay | OR curr smoke 1.48 (1.14-1.92) | OR curr smoke 1.12 (0.77-1.64) | Alcohol, literacy, knowledge of TB, missed doses, side effect, etc. |
| 2011 Dujaili | OR ever smoke 7.17 (2.76-18.62) | OR ever smoke 3.25 (1.01-10.45) | Age, sex, alcohol use, IVDU, history of chronic disease |
| 2011 Garcia-Garcia | NR | NR | NA |
| 2011 Maruza | NR | OR curr smoke 2.62 (1.31-5.26), past 1.07 (0.43-2.67) | Age, sex, education, CD4 count, HAART use |
| 2011 Tachfouti, 2013 Tachfouti | OR curr smoke 1.30 (1.10-1.50) | OR curr smoke 0.70 (0.50–1.10) | Age, sex, education, religion, income, alcohol, clinical form of TB |
| 2012 Lisha | NR | NR (higher risk with smoking) | Age, sex, income, weight loss, category of treatment, DM, |
| 2013 Reddy | OR curr smoke 10.33 (2.36-45.11) | NR | NA |
| 2013 Slama | NR | OR curr smoke 2.10 (1.07–4.14) | Age, sex, relapse, alcohol, insufficient explanation about disease |
| 2014 Cherkaoui | OR curr smoke 6.60 (3.40-12.20) | OR curr smoke 4.10 (1.50-10.90) | Age, retreatment, DOTS support, side effects, etc. |
| 2014 Choi | OR curr 1 pack/day 3.36 (1.75-6.46) | NR | NA |
| 2014 Ibrahim, 2015 Ibrahim | NR | NR | NA |
| 2015 Driessche | HR ever smoke 2.43 (1.43 – 4.13) | HR ever smoke 2.40 (1.13 – 5.11) | Age, sex, weight, CD4 count, ART, EPTB, alcohol, etc. |
| 2015 Leung | OR curr smoke 1.76 (1.55-2.01), past 1.16 (1.01-1.33) | NR | Age, sex, ethnicity, residence, alcohol, DM, HIV, etc. |
| 2015 Roy | OR curr smoke 1.50 (1.14-1.92) | OR curr smoke 1.22 (0.34-1.89) | Age, sex, education, employed, married, alcohol, etc. |
| 2016 Rathee | OR curr smoke 6.21 (1.61-23.91), past 1.50 (0.14–15.96) | NR | NA |
| 2019 Castro | OR curr smoke 1.86 (0.82, 4.18) | NR | NA |
| 2019 Paunikar | OR curr smoke 3.76 (1.23-11.49) | NR | NA |
| 2020 Bezerra | OR ever smoke 3.70 (1.12 – 13.14) | OR ever smoke 4.91 (1.08 – 22.32) | Sex, race, occupation, alcohol, type of TB, etc. |
| 2020 Khan | OR ever smoke 1.55 (1.29 - 1.86) | OR ever smoke 1.57 (1.31 - 1.89) | Age, sex, race, area, employment, alcohol, comorbidity, etc. |
| **OUTCOME 6: Failure (n=12)** | | | |
| 2002 Santha | OR current smoke 8.4 (1.0-388.0) | NR | NA |
| 2007 Wang | OR ever smoke 3.07 (0.28-34.11) | NR (not significant) | NA |
| 2010 Metanat | NR | NR | NA |
| 2011 Dujaili | OR ever smoke 7.49 (0.93-60.30) | OR ever 15.59 (0.59-308.69) | Age, sex, alcohol, drug use, history of chronic disease |
| 2011 Tachfouti, 2013 Tachfouti | OR curr smoke 2.37 (1.24–4.54) | OR curr smoke 2.25 (1.06–4.76) | Age, sex, education, religion, income, alcohol, TB form |
| 2013 Reddy | NR (significant) | NR | NA |
| 2014 Ibrahim, 2015 Ibrahim | NR | NR | NA |
| 2015 Khan | OR curr smoke 2.58 (1.32-5.03) | NR | NA |
| 2019 Aguilar | OR ever smoke 2.49 (1.34-4.63) | OR ever smoke 2.20 (1.10-4.70) | Age, sex, education, marital status, income, alcohol |
| 2019 Gupte, 2019 Thomas | NR | IRR past smoke 2.66 (1.41–4.90) | Age, sex, family income, HIV, DM, CXR cavity, smear |
| 2019 Ma | OR current smoke 2.08 (0.74-5.86), past 2.46 (1.29-4.69) | NR | NA |
| 2020 Serpoosh | OR curr smoke 2.58 (1.10-6.10) | OR curr smoke 1.26 (0.42-3.76) | Age, sex, drug use |
| **OUTCOME 7: Unsuccessful (n=28)** | | | |
| 2002 Leung, 2003 Leung | NR | NR | NA |
| 2009 Kherad | NR (not significant) | NR (not significant) | NA |
| 2011 Nik Mahdi | OR curr smoke 6.67 (2.70-16.50) | NR (not significant) | NA |
| 2011 Solliman | NR | NR | NA |
| 2012 Chiang | OR ever smoke 1.97 (1.22-3.17) | OR 1-20/day 1.64 (0.95-2.86), >20/day 4.35 (1.02-20.00) | Age, sex, concurrent disease, alcohol, adverse reaction, etc. |
| 2012 Tabarsi | OR curr smoke 0.27 (0.02-3.09) | NR | NA |
| 2013 Bonacci | NR | OR curr light smoke 1.46 (0.81-2.63), heavy 2.36 (1.1-5.05) | Age, sex, crowding, rural, new patient, HIV, etc. |
| 2013 Mnisi | OR curr smoke 1.56 (1.29-2.05) | NR | NA |
| 2014 Alo | OR curr smoke 0.79 (0.38-1.64) | NR | NA |
| 2014 Choi | OR curr smoke 1.14 (0.66-1.97) | NR | NA |
| 2014 Lucenko | RR curr smoke 1.20 (0.90-1.50) | NR | NA |
| 2014 Przybylski | OR curr smoke 1.01 (0.73–1.41) | NR | NA |
| 2015 Driessche | HR ever smoke 2.15 (1.45-3.20) | HE ever smoke 1.57 (1.00 – 2.47) | Age, sex, weight, CD4 count, ART, EPTB, alcohol, etc. |
| 2015 Gegia | RR current smoke 1.91 (1.25-2.94), SLT 0.95 (0.28-3.27) | RR current smoke 1.70 (1.00–2.90), SLT 1.63 (0.52–5.15) | Age, sex, income, prison history, alcohol, previous TB, etc. |
| 2015 Leung | OR curr smoke 1.26 (1.14-1.39), past 1.69 (1.54-1.85) | OR curr smoke 1.39 (1.22-1.56), past 1.37 (1.22-1.54) | Age, sex, ethnicity, residence, alcohol, DM, HIV, etc. |
| 2015 Liew | OR curr smoke 1.51 (1.41-1.61) | OR curr smoke 1.15 (1.03-1.28) | Age, sex, education, type of TB, BCG, DM, HIV, etc. |
| 2016 Veerakumar | OR current smoke 3.07 (1.43-6.59), SLT 1.75 (0.60-5.09) | OR current smoke 2.20 (0.91-5.50) | Age, sex, category of treatment, alcohol |
| 2017 Balian | OR curr smoke 2.05 (1.54-2.74) | OR curr smoke 1.60 (1.07–2.42) | Age, sex, sputum smear, anatomical site, HIV, alcohol |
| 2017 Jaber | OR curr smoke 1.57 (0.94–2.61) | OR curr smoke 1.47 (0.87–2.58) | Sex, education, employment, comorbidities |
| 2018 Cailleaux-Cezar | RR curr smoke 3.75 (1.15-12.22) | RR curr smoke 6.29 (1.57-25.21) | Age, sex, schooling, DM, comorbidities, alcohol, cavitation |
| 2018 Mukhtar | OR curr smoke 2.42 (1.33-4.38) | OR curr smoke 2.03 (1.04-3.93) | Age, residential area, DM, BMI |
| 2019 Gupta | OR ever smoke 13.00 (0.64-262.00), SLT 21.00 (1.65-266.90) | NR | NA |
| 2019 Gupte, 2019 Thomas | NR | IRR current smoke 2.03 (1.33-3.08), past 2.20 (1.27-3.81 | Age, sex, family income, HIV, DM, CXR cavity, smear |
| 2019 Ma | OR current smoke 1.60 (0.87-2.93), past 1.73 (1.21-2.49) | NR | NA |
| 2020 Sembiah | NR | NR | NA |
| 2020 Tok | OR curr smoke 1.44 (1.39-1.49) | OR curr smoke 1.08 (1.04-1.13) | Age, sex, nationality, rural, education, BCG, DM, HIV, etc. |
| 2021 de Vargas | OR curr smoke 2.89 (1.07-7.80) | NR | NA |
| 2021 Kassim | NR | OR curr smoke 5.72 (1.20-27.18) | BMI, DM, family size, lifestyle, health facilities, etc. |
| **OUTCOME 9: Delayed sputum conversion (n=25)** | | | |
| 2002 Leung, 2003 Leung | OR ever smoke 1.03 (0.73-1.45) | NR | NA |
| 2005 Abal | OR curr smoke 2.12 (0.94-4.77) | NR | NA |
| 2007 Babb | NR | RR curr smoke 1.96 (1.01-2.81) | Age, sex, education, disease extent, WBC count |
| 2007 Guler | NR | OR curr smoke 0.65 (0.25–1.70) | Age, sex, DM, cavity, radiology |
| 2010 Metanat | OR ever smoke 1.70 (0.39-7.32) | NR | NA |
| 2010 Siddiqui | NR | OR ever smoke 4.42 (1.23-15.90) | Age, sex |
| 2012 Feng | OR curr smoke 1.43 (0.99–2.08) | NR | NA |
| 2012 Visser | HR ever smoke 2.22 (1.22-4.00) | HR ever smoke 3.12 (0.98-10.00) | Age, sex, HIV, cavities, alcohol, BMI, haemoglobin, etc. |
| 2013 Maciel | OR curr smoke 2.28 (1.02–5.33), past 1.11 (0.45–2.76) | OR curr smoke 3.04 (1.35–6.83) | Age, sex, pre-treatment extent of disease |
| 2014 de Boer | OR curr smoke 5.60 (1.70-18.70) | OR curr smoke 6.90 (1.80-26.70) | Cavities, alcohol |
| 2014 Pefura-Yone | NR | OR current 7.26 (1.59-33.23) | Age, sex, TB duration, clinical signs, alcohol, BMI, HIV |
| 2015 Chuang | NR | HR current smoke 1.12 (1.03-1.39), past 0.83 (0.58-1.17) | Age, sex, BMI |
| 2015 Kanda | HR ever smoke 1.43 (0.92-2.22) | HR ever smoke 2.08 (1.06-4.00) | Age, sex, diabetes, alcohol use, TB disease severity |
| 2015 Leung | OR current smoke 2.01 (1.65-2.45), past 1.50 (1.23-1.83) | NR | NA |
| 2016 Ajili | OR curr smoke 2.57 (1.64-4.03) | NR (significant) | Cavities, non-observance to treatment |
| 2017 Altet | NR | OR curr smoke 2.78 (1.22-6.67) | Sex, DM, alcohol, HIV, COPD, body weight, drug use, etc. |
| 2017 Musteikiene | OR >20 PY vs. 1-20 PY 3.40 (0.99-11.60) | OR >20 PY vs. 1-20 PY 5.19 (0.93–29.10) | Age, sex, BMI, residence, occupation, education, health status, alcohol, etc. |
| 2018 Cailleaux-Cezar | RR curr smoke 2.87 (1.25-6.59) | RR curr smoke 3.58 (1.30-9.86) | Age, sex, schooling, comorbidities, alcohol, cavitation |
| 2019 Ma | OR current smoke 1.80 (0.85-3.78), past 2.06 (1.31-3.23) | NR | NA |
| 2019 Reimann (time to conversion) | NR | Mean days (higher with ever smoking, p<0.001) | Not clear |
| 2019 Wardani | OR curr smoke 5.93 (1.66-21.25) | OR curr smoke 7.46 (1.76-31.64) | Age, sex, education, occupation, social class, DM, etc. |
| 2020 Takasaka | HR ever smoke 1.81 (1.09-2.99) | HR ever smoke 0.87 (0.44–1.72) | Age, emphysema, cavity, time to detection, albumin, etc. |
| 2021 Asemahagn | OR curr smoke 2.20 (1.25-4.01) | OR curr smoke 1.96 (1.20-3.82) | Age, sex, residence, education, prior TB, HIV, DM, etc. |
| 2021 Bhatti | NR | OR ever smoke 2.10 (1.40-3.20) | Age, occupation, sputum grade, retreatment, CXR |
| 2021 Mokti | OR curr smoke 1.60 (1.20-2.20) | OR curr smoke 1.54 (1.10-2.10) | Age, nationality, rural, no DOTS supervisor, CXR, etc. |
| **OUTCOME 9: Treatment non-adherence (n=8)** | | | |
| 2005 Balbay | OR curr smoke 3.99 (1.90-8.35) | OR curr smoke 1.49 (1.12-1.98) | Age, sex, family history, new cases, cough, cavity, etc. |
| 2014 Ahmad | RR current smoke 1.27 (1.05-1.54), SLT 1.26 (1.08-1.47) | NR | NA |
| 2014 Ibrahim, 2015 Ibrahim | OR curr smoke 2.00 (1.11-3.67) | OR curr smoke 4.40 (1.49-7.95) | Age, sex, treatment site, education, alcohol, etc. |
| 2017 Tola | OR curr smoke 1.29 (0.69-2.42) | NR | NA |
| 2018 Madeira | OR ever smoke 1.99 (1.30-3.06) | OR ever smoke 1.72 (1.00-3.00) | Age, sex, race, education, income, drug use, HIV, DM |
| 2020 Ajema | OR curr smoke 27.9 (11.5-67.9) | OR curr smoke 11.7 (3.20-43.03) | Income, marital status, time to reach health facility, etc. |
| 2020 Khan | OR ever smoke 1.46 (1.18-1.80) | OR ever smoke 1.49 (1.21-1.84) | Age, sex, occupation, alcohol, IVDU, TB type, comorbidity |
| 2020 Pore | NR | NR | NA |
| **OUTCOME 10: Disease severity (n=5)** | | | |
| 2005 Altet-Gomez (length of hospitalisation) | NR | NR (higher with current smoking, p<0.001) | Age, sex, alcohol, infection site |
| 2019 Ma (cavitation) | OR current smoke 2.80 (0.96-8.18), past 1.52 (0.68-3.43) | NR | NA |
| 2019 Nakao (cavitation) | OR ever smoke 2.88 (1.16-7.13) | NR | NA |
| 2019 Reimann (length of hospitalisation) | NR | Mean days (higher with ever smoking, p<0.001) | Not clear |
| 2021 Cao (rehospitalisation) | OR curr smoke 2.34 (1.60-3.44) | OR curr smoke 2.15 (1.37-3.40) | Age, sex, residence, insurance, comorbidities, etc. |
| **OUTCOME 11: Developing drug resistance (n=1)** | | | |
| 2019 Sharma | OR curr smoke 2.33 (1.65-3.30) | OR curr smoke 2.56 (1.19-3.26) | Age, education, occupation, HIV, alcohol, migration, etc. |

# e-Figure 1: Forest plots of subgroup and sensitivity analyses for TB recurrence/relapse

## Ever-smoking – subgroup, study design


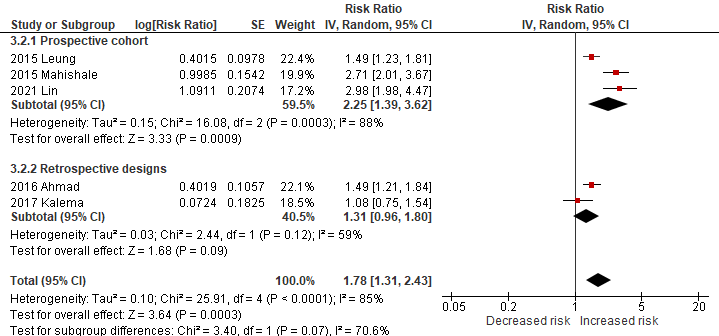


## Ever-smoking – subgroup, study quality


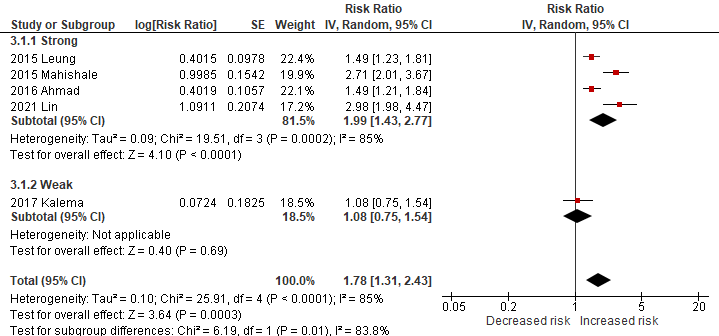


## Ever-smoking – subgroup, comorbidities


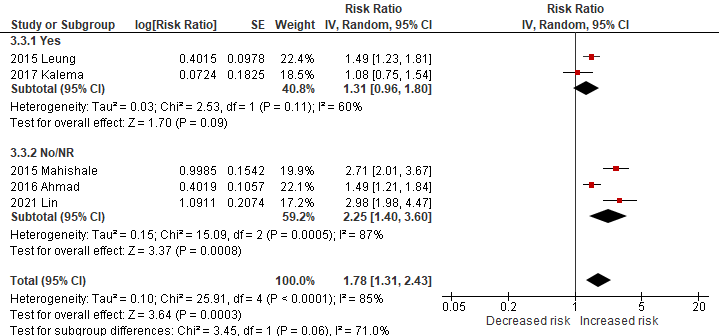


## Ever-smoking – sensitivity, removing DR-TB studies


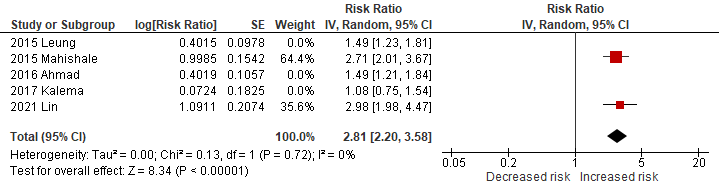


## Ever-smoking – sensitivity, removing retreatment-TB studies


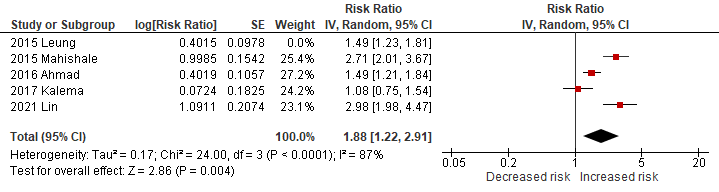


## Current-smoking – subgroup, study design


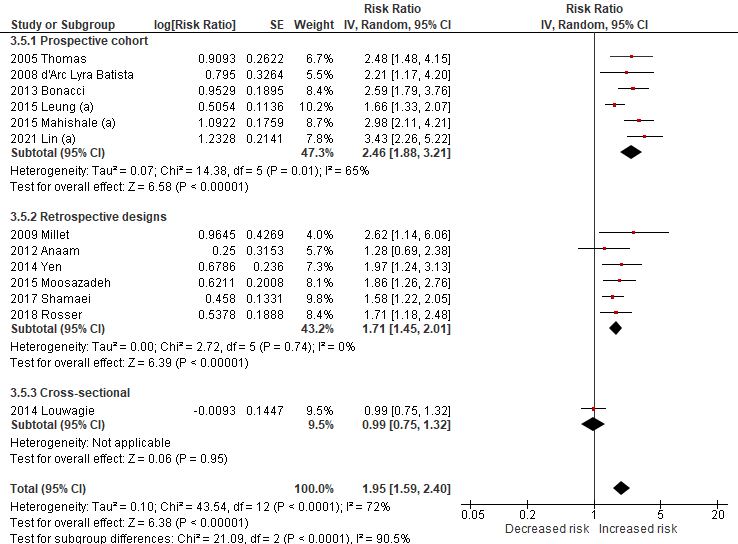


## Current-smoking – subgroup, study quality


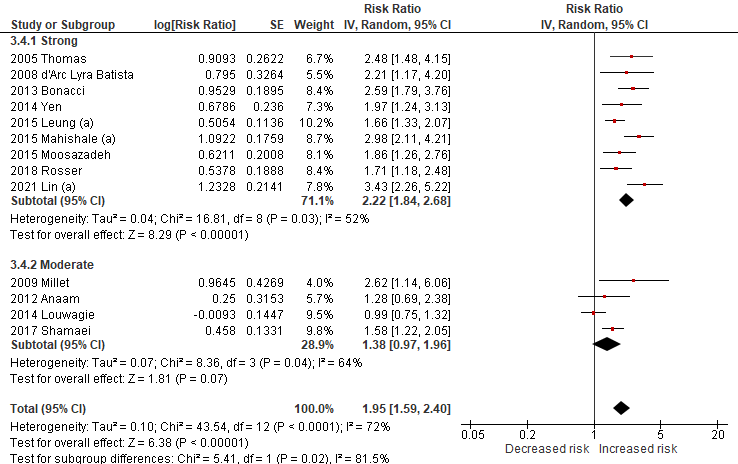


## Current-smoking – subgroup, comorbidities


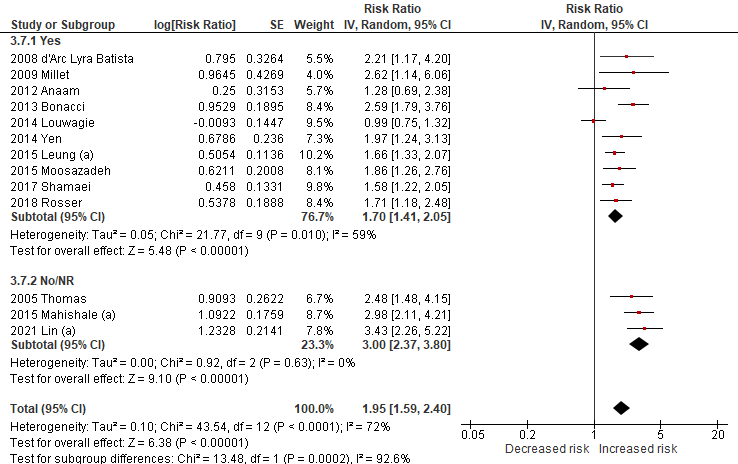


## Current-smoking – sensitivity, removing DR-TB studies


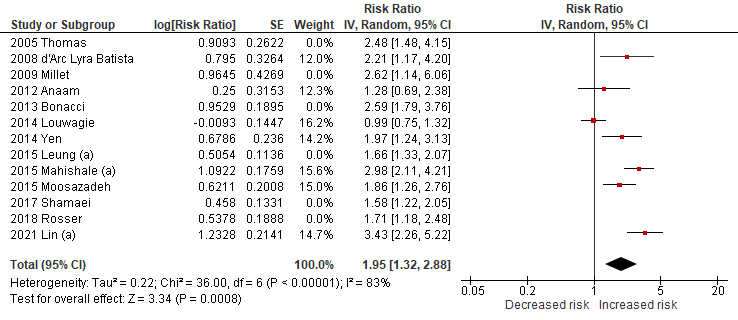


## Current-smoking – sensitivity, removing retreatment-TB studies


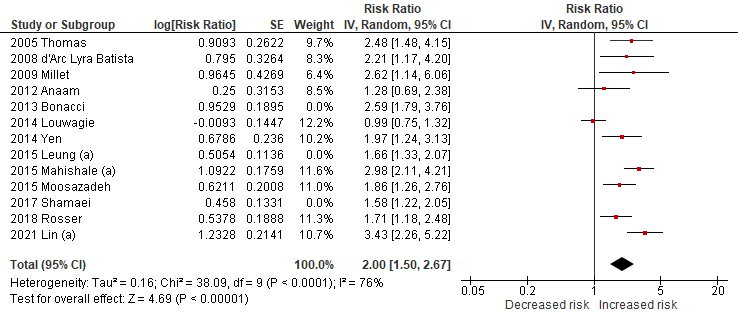


## Past-smoking – subgroup, comorbidities


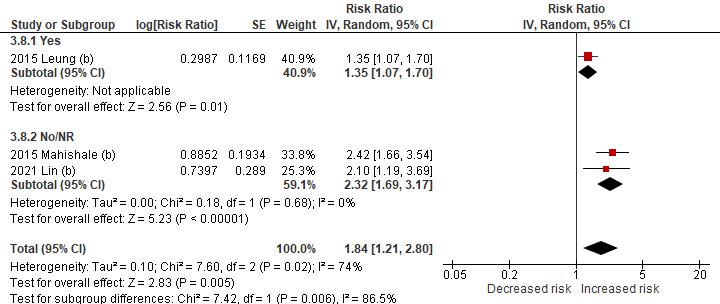


## Past-smoking – sensitivity, removing DR-TB studies


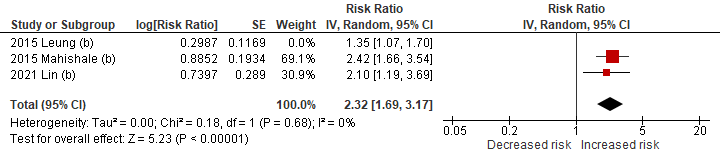


## Past-smoking – sensitivity, removing retreatment-TB studies


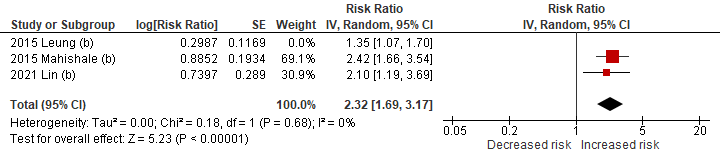


# e-Figure 2: Publication bias for TB recurrence/relapse

## Ever-smoking – funnel plot


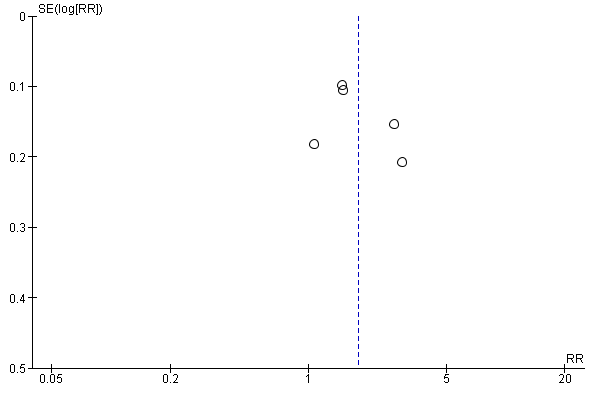


## Current-smoking – funnel plot


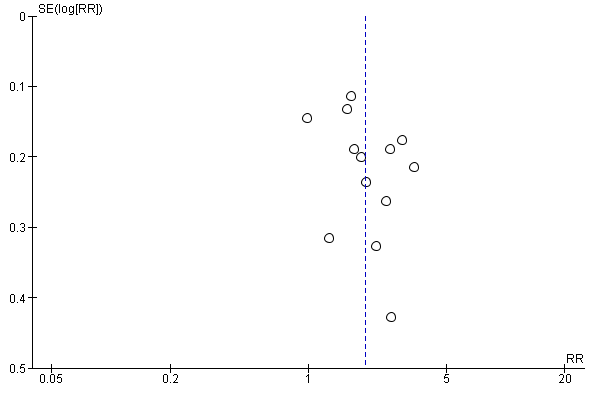


## Past-smoking – funnel plot


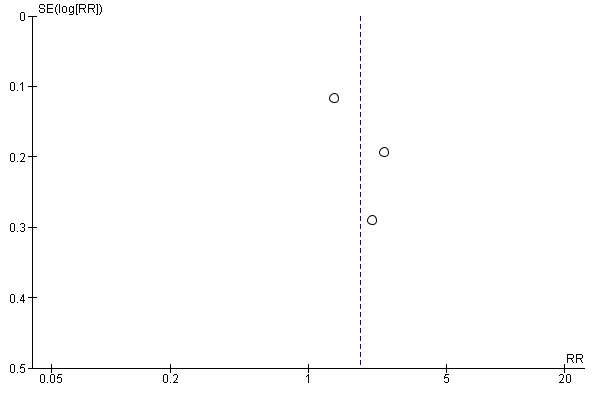


# e-Figure 3: GRADE for TB recurrence/relapse

## Ever-smoking

|  | | | Rating | Adjustment to score |
| --- | --- | --- | --- | --- |
| **Quality assessment** | No. of studies and Starting score | | 5 observational | 2 |
|  | Factors decreasing confidence | Limitation in study design | Not serious^1^ | 0 |
|  |  | Inconsistency | Serious^2^ | -1 |
|  |  | Indirectness | Not serious | 0 |
|  |  | Imprecision | Not serious | 0 |
|  |  | Publication bias | Not serious | 0 |
|  | Factors increasing confidence | Large effect | Weak evidence | 0 |
|  |  | Dose-response | Weak evidence | 0 |
|  |  | Mitigated bias and confounding | Weak evidence | 0 |
|  | Final GRADE score of quality of evidence | | | 1 |
| **Summary** | Quality of evidence | | | Very Low  ⊕ |
| ^1^ Four of five studies were rated ‘strong’; 1 was ‘weak’  ^2^ Strong evidence of heterogeneity found, explained to some extent by subgroup analyses | | | | |

## Current-smoking

|  | | | Rating | Adjustment to score |
| --- | --- | --- | --- | --- |
| **Quality assessment** | No. of studies and Starting score | | 13 observational | 2 |
|  | Factors decreasing confidence | Limitation in study design | Not serious^1^ | 0 |
|  |  | Inconsistency | Serious^2^ | -1 |
|  |  | Indirectness | Not serious | 0 |
|  |  | Imprecision | Not serious | 0 |
|  |  | Publication bias | Not serious | 0 |
|  | Factors increasing confidence | Large effect | Weak evidence | 0 |
|  |  | Dose-response | Weak evidence | 0 |
|  |  | Mitigated bias and confounding | Weak evidence | 0 |
|  | Final GRADE score of quality of evidence | | | 1 |
| **Summary** | Quality of evidence | | | Very Low  ⊕ |
| ^1^ All studies were rated ‘strong’ or ‘moderate’  ^2^ Strong evidence of heterogeneity found, explained to some extent by subgroup analyses | | | | |

## Past-smoking

|  | | | Rating | Adjustment to score |
| --- | --- | --- | --- | --- |
| **Quality assessment** | No. of studies and Starting score | | 3 observational | 2 |
|  | Factors decreasing confidence | Limitation in study design | Not serious^1^ | 0 |
|  |  | Inconsistency | Serious^2^ | -1 |
|  |  | Indirectness | Not serious | 0 |
|  |  | Imprecision | Not serious | 0 |
|  |  | Publication bias | Not serious | 0 |
|  | Factors increasing confidence | Large effect | Weak evidence | 0 |
|  |  | Dose-response | Weak evidence | 0 |
|  |  | Mitigated bias and confounding | Weak evidence | 0 |
|  | Final GRADE score of quality of evidence | | | 1 |
| **Summary** | Quality of evidence | | | Very Low  ⊕ |
| ^1^ All studies were rated ‘strong’  ^2^ Strong evidence of heterogeneity found, explained to some extent by subgroup analyses | | | | |

# e-Figure 4: Forest plots of subgroup and sensitivity analyses for Mortality during TB treatment

## Ever-smoking – sensitivity, removing DR-TB studies (no studies removed)


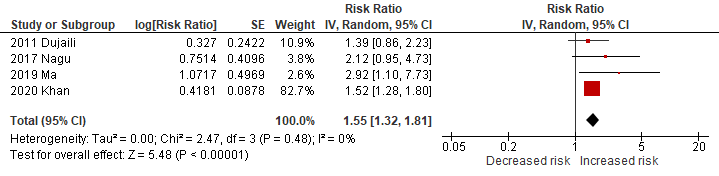


## Ever-smoking – sensitivity, removing retreatment-TB studies


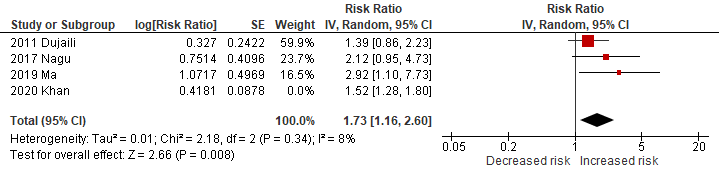


## Current-smoking – subgroup, study design


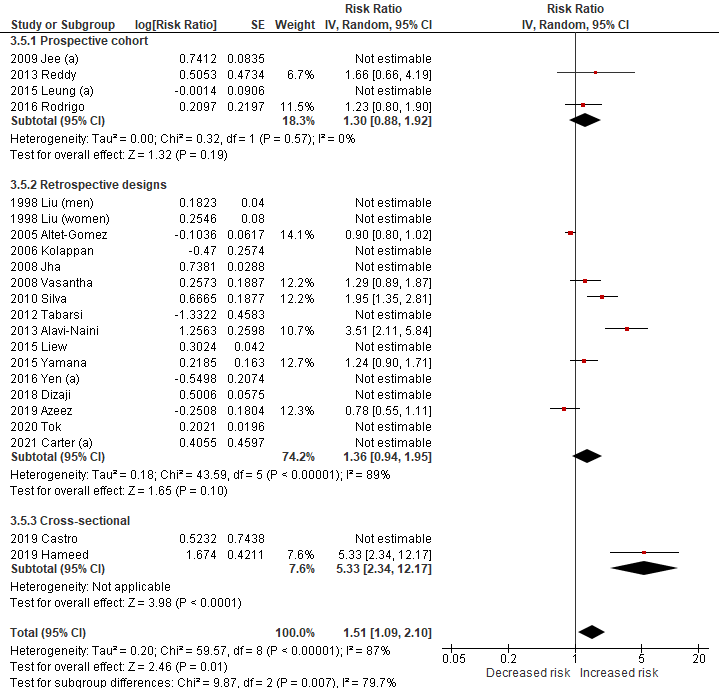


## Current-smoking – subgroup, study quality


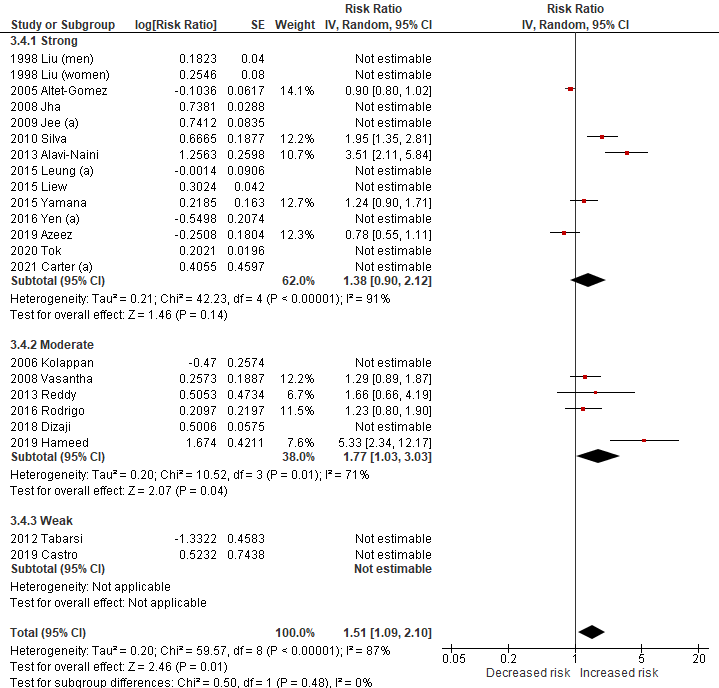


## Current-smoking – subgroup, comorbidities


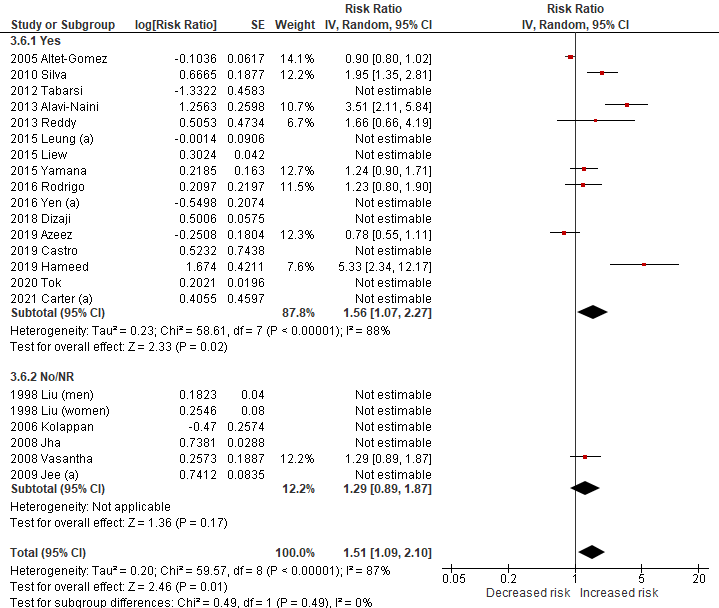


## Current-smoking – sensitivity, removing DR-TB studies


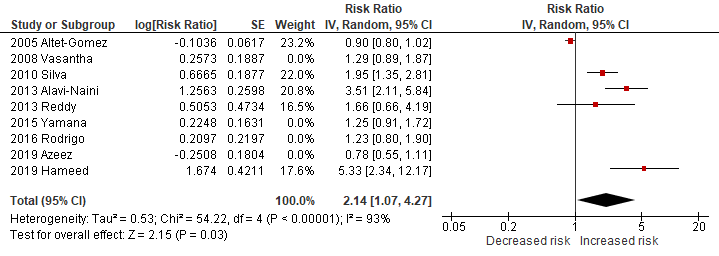


## Current-smoking – sensitivity, removing retreatment-TB studies


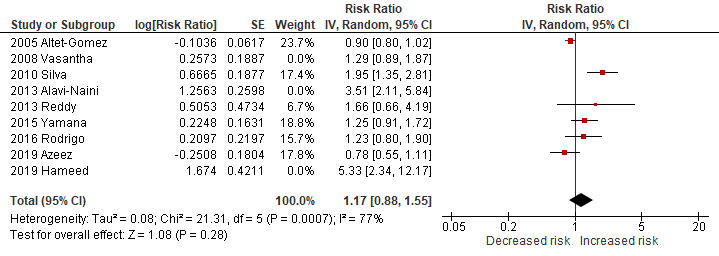


# e-Figure 5: Publication bias for Mortality during TB treatment

## Ever-smoking – funnel plot


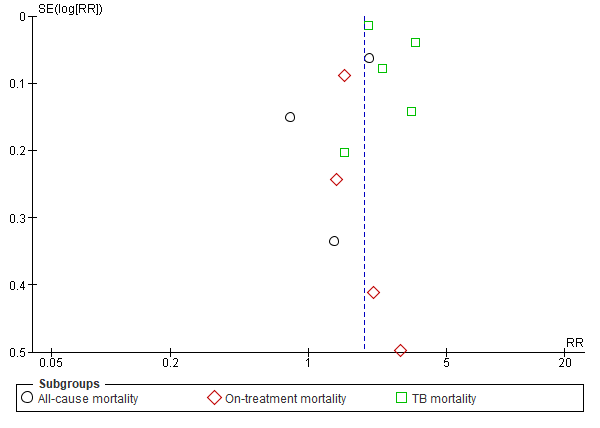


## Current-smoking – funnel plot


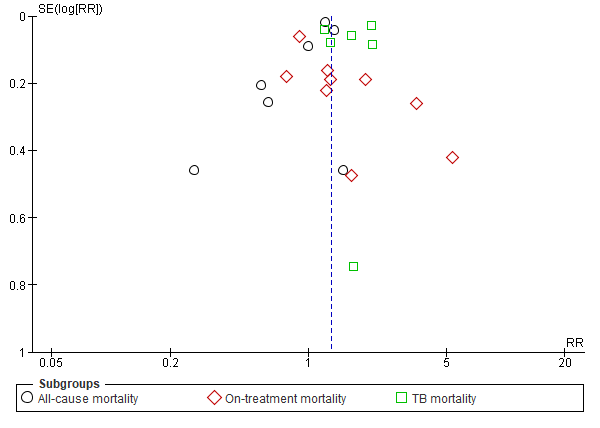


# e-Figure 6: GRADE for Mortality during TB treatment

## Ever-smoking

|  | | | Rating | Adjustment to score |
| --- | --- | --- | --- | --- |
| **Quality assessment** | No. of studies and Starting score | | 4 observational | 2 |
|  | Factors decreasing confidence | Limitation in study design | Not serious^1^ | 0 |
|  |  | Inconsistency | Not serious^2^ | 0 |
|  |  | Indirectness | Not serious | 0 |
|  |  | Imprecision | Not serious | 0 |
|  |  | Publication bias | Not serious | 0 |
|  | Factors increasing confidence | Large effect | Weak evidence | 0 |
|  |  | Dose-response | Weak evidence | 0 |
|  |  | Mitigated bias and confounding | Weak evidence | 0 |
|  | Final GRADE score of quality of evidence | | | 2 |
| **Summary** | Quality of evidence | | | Low  ⊕⊕ |
| ^1^ All studies were rated ‘strong’ or ‘moderate’  ^2^ No heterogeneity found | | | | |

## Current-smoking

|  | | | Rating | Adjustment to score |
| --- | --- | --- | --- | --- |
| **Quality assessment** | No. of studies and Starting score | | 9 observational | 2 |
|  | Factors decreasing confidence | Limitation in study design | Not serious^1^ | 0 |
|  |  | Inconsistency | Not Serious^2^ | 0 |
|  |  | Indirectness | None serious | 0 |
|  |  | Imprecision | None serious | 0 |
|  |  | Publication bias | None serious | 0 |
|  | Factors increasing confidence | Large effect | Weak evidence | 0 |
|  |  | Dose-response | Weak evidence | 0 |
|  |  | Mitigated bias and confounding | Weak evidence | 0 |
|  | Final GRADE score of quality of evidence | | | 2 |
| **Summary** | Quality of evidence | | | Low  ⊕⊕ |
| ^1^ All studies were rated ‘strong’ or ‘moderate’  ^2^ Strong evidence of heterogeneity found, but largely explained by study design | | | | |
